# Phylogenetic overview of *Aureoboletus* (Boletaceae, Boletales), with descriptions of six new species from China

**DOI:** 10.3897/mycokeys.61.47520

**Published:** 2019-12-17

**Authors:** Ming Zhang, Tai-Hui Li, Chao-Qun Wang, Nian-Kai Zeng, Wang-Qiu Deng

**Affiliations:** 1 State Key Laboratory of Applied Microbiology Southern China, Guangdong Provincial Key Laboratory of Microbial Culture Collection and Application, Guangdong Institute of Microbiology, Guangdong Academy of Sciences, Guangzhou 510070, China; 2 Department of Pharmacy, Hainan Medical University, Haikou 571101, China

**Keywords:** Boletes, molecular phylogeny, morphology, species identification, taxonomy

## Abstract

In this study, species relationships of the genus *Aureoboletus* were studied, based on both morphological characteristics and a four-gene (nrLSU, *tef1-a*, *rpb1* and *rpb2*) phylogenetic inference. Thirty-five species of the genus have been revealed worldwide, forming eight major clades in the phylogenetic tree, of which twenty-four species have been found in China, including six new species: *A. glutinosus*, *A. griseorufescens*, *A. raphanaceus*, *A. sinobadius*, *A. solus*, *A. velutipes* and a new combination *A. miniatoaurantiacus* (Bi & Loh) Ming Zhang, N.K. Zeng & T.H. Li proposed here. A key to 24 known Chinese species has been provided.

## Introduction

*Aureoboletus* Pouzar was circumscribed in 1957, based on the type species *A. gentilis* (Quél.) Pouzar (Pouzar 1957). It was characterised by its slimy basidiomata, glabrous to subglabrous pileus and golden yellow hymenophore unchanging when dry (Quélet 1884; Saccardo 1888; Pouzar 1957). To date, 35 species have been described worldwide, 15 of which were originally described in China (Patouillard 1895; Shi and Liu 2013; Zhang et al. 2014, 2015a, b, 2017; Zeng et al. 2015; Li et al. 2016; Wu et al. 2016; Fang et al. 2019). *Aureoboletus* species can be found in tropical, subtropical and temperate regions of different continents, but most known species appear to exist in Asia and North America. Interestingly, they are strongly implicated as symbionts with an array of ectotrophic plants of the Fagaceae and Pinaceae families (Pouzar 1957; Yang et al. 2003; Klofac 2010; Shi and Liu 2013; Halling et al. 2015; Zeng et al. 2015; Wu et al. 2016; Zhang et al. 2017).

The establishment and acceptance of the genus *Aureoboletus* has a long history. Xerocomus section AuriporiSinger (1942) was established to accommodate *Aureoboletus*-like taxa. Later, *Auripori* species were transferred to the genus *Pulveroboletus* Murrill (Singer 1947). For a long time, the genus *Aureoboletus* was not accepted as an independent genus by some mycologists (Smith and Thiers 1971; Corner 1972; Singer 1986; Both 1993; Bessette et al. 2000; Šutara 2005) and species with viscid basidiomata and vivid yellow hymenophores were variously placed in genera *Boletellus* Murrill, *Boletus* L., *Pulveroboletus* and *Xerocomus* Quél. (Singer 1942, 1947, 1986; Smith and Thiers 1971; Corner 1972; Both 1993; Bessette et al. 2000); However, *Aureoboletus* was accepted as an independent genus by other mycologists and the features of the genus were redefined (Watling 1965; Watling 2008; Hongo 1973; Zang 1993; Šutara 2008; Klofac 2010). A world-wide survey of the genus, based on morphological characteristics, was conducted and a key was designed to aid in the identification of 11 global *Aureoboletus* species (Klofac 2010).

Recently, broad-scale molecular phylogenetic studies have been used to investigate phylogenetic relationships amongst the genera and species in Boletes. *Aureoboletus* was strongly supported as a genus in the Boletaceae, subfamily Xerocomoideae and has been shown to be closely related to *Boletellus*, *Hemileccinum* Šutara, *Heimioporus* E. Horak, *Xerocomus* etc. (Binder 1999; Binder and Hibbett 2006; Nuhn et al. 2013; Wu et al. 2014, 2016). The genus *Sinoboletus* M. Zang, originally described in south-western China, was proven to be a synonym of *Aureoboletus* (Zang 1992; Wu et al. 2014); *Boletellus projectellus* (Murrill) Singer, *B. mirabilis* (Murrill) Singer, *B. russellii* (Frost) E.-J. Gilbert and *Pulveroboletus auriflammeus* (Berk. & M.A. Curtis) Singer) were transferred into the genus *Aureoboletus*, based on both morphological and molecular data (Halling et al. 2015; Wu et al. 2016).

Numerous *Aureoboletus* specimens have been recently obtained in China, increasing the species diversity of *Aureoboletus*. In this study, the species richness and phylogenetic relationships were re-evaluated, based on detailed morphological observations and a four-gene phylogenetic inference. The aims were to 1) evaluate the phylogenetic relationships within the genus; 2) redefine the characteristics of *Aureoboletus*; 3) elucidate the species diversity of *Aureoboletus* in China; 4) describe the newly discovered species.

## Materials and methods

### Morphological studies

Photographs and records of basidiomata were obtained in the field. Specimens were dried in an electric drier and finally deposited in the Fungarium of Guangdong Institute of Microbiology (**GDGM**) or the Fungal Herbarium of Hainan Medical University (**FHMU**), Haikou City, Hainan Province, China. Descriptions of macro-morphological characters and habitats were obtained with photographs and field notes. Colours were described in general terms with serial numbers, for example, reddish-brown (9D8–9E8), following Kornerup and Wanscher (1978). Micro-morphological features were observed from dried materials after sectioning and mounting in 5% potassium hydroxide (KOH) solution and 1% Congo Red or Melzer’s reagent under a light microscope (Olympus BX51, Tokyo, Japan). For basidiospore descriptions, an abbreviation [n/m/p] denotes n spores measured from m basidiomata of p collections; a notation (a–)b–c(–d) describes basidiospore dimensions, where the range b–c represented 90% or more of the measured values and ‘a’ and ‘d’ were the extreme values; Q referred to the length/width ratio of an individual basidiospore and Qm referred to the average Q value of all basidiospores ± sample standard deviation. All line-drawings of microstructures were made, based on rehydrated materials.

### DNA extraction, PCR amplification and sequencing

Genomic DNA was extracted from the voucher specimens using the Sangon Fungus Genomic DNA Extraction kit (Sangon Biotech Co. Ltd., Shanghai, China), according to the manufacturer’s instructions. Primer pairs LR0R/LR5 or LR0R/LR7 (Vilgalys and Hester 1990), EF1-B-F1/EF1-B-R, RPB1-B-F/RPB1-B-R and RPB2-B-F1/RPB2-B-R (Wu et al. 2014) were used for the amplification of the large subunit nuclear ribosomal RNA (nrLSU) region, the translation elongation factor 1-alpha subunit (*tef1-a*), the largest subunit of RNA polymerase II (*rpb1*) and the second largest subunit of RNA polymerase II (*rpb2*), respectively. Polymerase Chain Reaction was performed in a total volume of 25 μl containing 1 μl template DNA, 9.5 μl distilled water, 1 μl of each primer and 12.5 μl PCR mix [DreamTaqtm Green PCR Master Mix (2×), Fermentas]. Amplification reactions were performed in a Tprofessional Standard thermocycler (Biometra, Göttingen, Germany) under the following conditions: at 95 °C for 4 min, then 35 cycles of denaturation at 95 °C for 60 s, annealing at 53 °C (LSU) /55 °C (*tef1-a*, *rpb1* and *rpb2*) for 60 s and extension at 72 °C for 80 s, with a final extension at 72 °C for 8 min. The PCR products were electrophoresed on 1% agarose gels with known standard DNA markers and sequences were performed on an ABI Prism 3730 Genetic Analyzer (PE Applied Biosystems, Foster, CA, USA) at Beijing Genomic Institute (BGI) using the same primers. The raw sequences were assembled with SeqMan implemented in Lasergene v7.1 (DNASTAR Inc., USA). The assembled sequences of the specimens were submitted to GenBank.

### Phylogenetic analyses

Newly generated sequences and related sequences downloaded from GenBank were used to reconstruct phylogenetic trees. Detailed information of samples, including species name, voucher, locality, GenBank accession numbers and references, are given in Table 1. Four sequence datasets (nrLSU, *tef1-a*, *rpb1* and *rpb2*) were separately aligned with MAFFT v6.853 using the E-INS-i strategy (Katoh et al. 2002) and examined in Bioedit v7.0.9 (Hall 1999). The four datasets were analysed independently using the Maximum Likelihood (ML) method to detect the topologies of the four genes. Since no significant incongruence was detected (BS > 70%), the four single-gene alignments were concatenated using Phyutility 2.2 (Smith and Dunn 2008). Missing fragments of some gene markers of several specimens were coded as missing data, intron regions of protein-coding genes were retained in the final analyses and the ambiguously aligned regions were detected and excluded with Gblocks (Castresana 2000).

**Table 1.** Information of samples used in this study.

Taxon	Voucher	Country	LSU	tef1	rpb1	rpb2	Reference
*A. auriflammeus*	DD973	USA	AY612818	_	_	_	GenBank
*A. auriporus*	MAN020	Costa Rica	JQ003659	_	_	_	Neves et al. 2012
	BDCR0431	Costa Rica	HQ161871	_	HQ161840	_	Dentinger et al. 2010
A. cf. auriporus	GDGM 44404	USA	MN410705	_	_	_	This study
*A. catenarius*	GDGM 45142	China	MN204514	_	MN473157	_	This study
	HKAS54463	China	KT990509	KT990710	KT990890	KT990348	Wu et al. 2016
	HKAS54467	China	KT990510	KT990711	_	KT990349	Wu et al. 2016
*A. citriniporus*	REH8719	USA	KF030298	_	_	_	Nuhn et al. 2013
*A. clavatus*	GDGM42992	China	MK123462	MK165847	_	_	Zeng et al. 2015
	GDGM42962	China	KR052045	MK165846	KR052056	_	Zeng et al. 2015
	GDGM42963	China	KR052046	KR052054	KR052057	_	Zeng et al. 2015
	GDGM42984	China	KR052047	KR052055	_	_	Zeng et al. 2015
*A. duplicatoporus*	GDGM 49451	China	MN204515	_	MN473160	_	This study
	GDGM 53135	China	MN204517	MN549677	MN473167	_	This study
	GDGM 53134	China	MN204516	_	MN473166	MN549707	This study
	GDGM 52898	China	MN410708	_	MN473164	_	This study
	GDGM 53181	China	MN204518	MN549669	MN473168	_	This study
	GDGM 71293	China	MN204519	_	MN473173	_	This study
	GDGM 71724	China	MN204520	_	MN473175	_	This study
	HKAS50498	China	KF112361	KF112230	KF112561	KF112754	Wu et al. 2016
	HKAS63009	China	KT990511	KT990712	KT990891	KT990350	Wu et al. 2016
	HKAS83115	China	KT990512	KT990713	KT990892	KT990351	Wu et al. 2016
	GDGM45133	China	MK123455	MK165834	_	MN549697	This study
	GDGM52889	China	MK123456	MK165835	MN473163	_	This study
*A. formosus*	GDGM44441	China	KT291749	KT291744	MN473152	KT291751	Zhang et al. 2015
	GDGM44444	China	KT291750	MK165833	MN473153	KT291752	Zhang et al. 2015
*A. gentilis*	Pug1	Germany	DQ534635	KF030399	_	_	Nuhn et al. 2013
	MG372a	Italy	KF112344	KF134014	KF112557	KF112741	Wu et al. 2014
*A. glutinosus*	GDGM 55717	China	MN204522	_	_	_	This study
	GDGM 45927	China	MN204521	_	-	MN549699	This study
	GDGM44476	China	MH670254	MH700192	_	MH700228	This study
	GDGM44477	China	MH670255	MH700205	_	MH700229	This study
	GDGM44479	China	MH670256	MH700204	_	MH700230	This study
	GDGM44733	China	MH670257	MH700203	_	MH700231	This study
	GDGM44821	China	MH670258	_	_	MH700232	This study
*A. griseolorufescens*	GDGM28490	China	MH670278	_	_	MH700241	This study
	ZhangM131	China	MH670279	_	MH700220	MH700242	This study
*A. innixus*	136/98	USA	DQ534639	_	_	_	Binder and Hibbett 2007
	MB03-104	USA	KF030239	KF030400	_	_	Nuhn et al. 2013
	136	USA	KF030240	_	_	_	Nuhn et al. 2013
*A. liquidus*	TNS:F-39710	Japan	AB972886	_	_	_	Terashima et al. 2016
	TNS:F-52265	Japan	AB972884	_	_	_	Terashima et al. 2016
	TNS:F-52267	Japan	AB972885	_	_	_	Terashima et al. 2016
*A. longicollis*	GDGM 70547	China	MN204526	_	MN473172	_	This study
	GDGM 75292	China	MN204527	_	MN473179	_	This study
	GDGM 49735	China	MN204525	_	MN473161	_	This study
	GDGM 43502	China	MN204524	_	MN473150	MN549688	This study
	ZhangM56	China	MN204528	_	MN473187	_	This study
	GDGM43239	China	MK123459	MK165843	MN473147	_	This study
	HKAS80127	China	KT990515	KT990719	_	_	Zeng et al. 2015
	GDGM42849	China	KR052051	_	KR052058	_	Zeng et al. 2015
	HKAS53398	China	KF112376	KF112238	KF112625	KF112755	Zeng et al. 2015
	HKAS84679	China	KT990514	KT990718	_	KT990356	Zeng et al. 2015
	HKAS80489	China	KT990523	KT990727	_	KT990364	Zeng et al. 2015
	GDGM44734	China	MK123458	MK165842	MN473155	MN549692	This study
	GDGM53336	China	MK123460	MK165844	MN473170	MN5497018	This study
	GDGM44739	China	MK123461	MK165845	MN473156	MN549693	This study
*A. marronius*	GDGM43288	China	KJ488958	KT291746	_	KT291753	Zhang et al. 2014
*A. miniatoaurantiacus*	GDGM 75495	China	MN204533	_	MN473181	MN549711	This study
	GDGM 53350	China	MN204532	MN549678	MN473171	MN549709	This study
	GDGM 43437	China	MN204530	_	MN473149	MN549687	This study
	GDGM 43282	China	MN204529	MN549671	MN473148	MN549686	This study
	GDGM 44727	China	MN204531	_	MN473154	MN549691	This study
	GDGM53501	China	MH670262	MH700199	MH700217	_	This study
	Zeng1625	China	MH670263	_	_	_	This study
	Zeng1294	China	MH670264	MH700198	_	_	This study
	Zeng1323	China	MH670265	MH700197	_	_	This study
	Zeng1339	China	MH670266	MH700196	_	_	This study
	Zeng664	China	MH670267	MH700195	_	_	This study
	HKAS59694	China	KT990513	KT990714	KT990893	KT990352	Wu et al. 2016
	GDGM42855	China	MH670259	MH700202	MH700214	MH700233	This study
	GDGM53274	China	MH670260	MH700201	MH700215	MH700234	This study
	GDGM52888	China	MH670261	MH700200	MH700216	MH700235	This study
*A. mirabilis*	REH9765	USA	KP327661	KP327661	_	_	Halling et al. 2015
	CBS-136.60	Germany	AF050652	_	_	_	Binder and Fischer 1997
	HKAS57776	China	KF112360	KF112229	KF112624	KF112743	Wu et al. 2014
	REH8717	USA	KF030299	_	_	_	Nuhn et al. 2013
*A. moravicus*	Xle1	Germany	_	KF030403	_	_	Nuhn et al. 2013
	MG374a	Italy	KF112421	KF112232	KF112559	KF112745	Wu et al. 2014
*A. nephrosporus*	HKAS67931	China	KT990516	KT990720	KT99089	KT990357	Wu et al. 2016
	HKAS74929	China	KT990517	KT990721	KT990896	KT990358	Wu et al. 2016
*A. novoguineensis*	K-A/7	Japan	DQ534637	_	_	_	Binder and Hibbett 2007
*A. projectellus*	MB-03-118	USA	NG027638	_	_	_	GenBank
	NYBG13392	USA	KP327622	KP327675	_	_	Halling et al. 2015
	Sn2Hor	USA	KF030300	_	_	_	Nuhn et al. 2013
	NYBG13393	USA	KP327623	KP327676	_	_	Halling et al. 2015
	ID-713	USA	DQ534582	AY879116	AY788850	AY787218	Binder and Hibbett 2007
*A. quercus-spinosae*	GDGM 43757	China	KY039966	MK165839	KY039962	KY039957	Zhang et al. 2017
	GDGM43757	China	KY039966	MK165839	KY039962	KY039957	Zhang et al. 2017
	GDGM43755	China	KY039967	MK165836	KY039963	KY039958	Zhang et al. 2017
	GDGM43758	China	KY039968	MK165837	KY039964	KY039959	Zhang et al. 2017
	GDGM43786	China	KY039969	MK165838	KY039965	KY039960	Zhang et al. 2017
*A. raphanaceus*	GDGM 45966	China	MN204536	MN549673	_	MN549700	This study
	GDGM 52266	China	MN204538	MN549674	_	MN549702	This study
	GDGM 45911	China	MN204535	_	_	MN549698	This study
	GDGM 52908	China	MN204539	MN549675	_	_	This study
	GDGM 49634	China	MN204537	_	_	MN549701	This study
	GDGM 53127	China	MN204540	MN549676	MN473165	MN549706	This study
	GDGM 75476	China	MN204541	_	MN473166	MN549707	This study
	GDGM 42937	China	MN204534	_	MN473146	MN549685	This study
	GDGM52543	China	MH670271	_	_	_	This study
	GDGM44832	China	MH670268	MH700194	MH700218	MH700236	This study
	GDGM50266	China	MH670269	_	_	MH700237	This study
	GDGM46333	China	MH670270	_	_	MH700238	This study
	GDGM52590	China	MH670272	MH700193	MH700219	MN549704	This study
*A. roxanae*	DS626-7	USA	KF030311	KF030402	KF030381	_	Nuhn et al. 2013
*A. rubellus*	GDGM52382	China	MH670273	_	_	MH700239	This study
	GDGM52367	China	MH670274	_	_	MH700240	This study
*A. shichianus*	HKAS43373	China	AY647211	_	_	_	GenBank
	HKAS76852	China	KF112419	KF112237	KF112562	KF112756	Wu et al. 2014
*A. sinobadius*	GDGM75499	China	MN204551	_	MN473182	_	This study
	GDGM 70666	China	MN204547	_	_	_	This study
	GDGM 49747	China	MN204546	_	_	_	This study
	GDGM 49670	China	MN204545	_	_	_	This study
	GDGM 71932	China	MN204548	_	MN473176	_	This study
*A. sinobadius*	GDGM 49482	China	MN204544	_	_	_	This study
	GDGM 72253	China	MN204549	_	MN473177	_	This study
	GDGM 49432	China	MN204543	_	MN473159	_	This study
	GDGM 75477	China	MN204550	_	MN473180	_	This study
	GDGM44473	China	MH670250	MH700189	MH700211	_	This study
	GDGM43275	China	MH6702464	MH700185	MH700208	MH700221	This study
	GDGM44732	China	MH670247	MH700186	MH700207	MH700222	This study
	GDGM44730	China	MH670248	MH700187	MH700209	MH700223	This study
	GDGM44736	China	MH670249	MH700188	MH700210	MH700224	This study
*A. solus*	GDGM46222	China	MH670275	_	_	_	This study
	GDGM44759	China	MH670276	MH700206	_	_	This study
	GDGM42822	China	MH670277	_	_	_	This study
	GDGM 49600	China	MN410707	_	_	_	This study
	GDGM 49404	China	MN204553	_	_	_	This study
	GDGM 46807	China	MN204552	_	_	_	This study
	GDGM 72441	China	MN204555	_	_	_	This study
	GDGM 70342	China	MN204554	_	_	_	This study
*Aureoboletus* sp.	GDGM 49259	China	MN410706	_	MN473158	_	This study
	GDGM 71707	China	MN204556	_	MN473174	MN549710	This study
	GDGM 75305	China	MN204513	_	_	_	This study
	GDGM 70474	China	MN204511	_	_	_	This study
	GDGM 72473	China	MN204512	_	_	_	This study
	GDGM 44470	China	MN204509	MN549672	_	MN549680	This study
	GDGM 52298	China	MN204523	_	MN473162	MN549703	This study
	LAM-0466	Malaysia	KY091058	_	_	_	GenBank
	HKAS53458	China	KF112456	KF112231	KF112558	KF112742	This study
	GDGM44829	China	KY039970	_	_	KY039961	This study
	GDGM44831	China	KY039971	MK165840	_	MN549696	This study
	GDGM44469	China	KP319028	MK165841	_	MN549690	This study
*A. tenuis*	GDGM42601	China	KF534789	KT291745	_	KT291754	Zhang et al. 2013
*A. thibetanus*	HKAS76655	China	KF112420	KF112236	KF112626	KF112752	Wu et al. 2014
	GDGM43283	China	KJ907380	KT291747	_	KT291755	Zhang et al. 2014
	GDGM43284	China	KJ90738	KT291748	_	KT291756	Zhang et al. 2014
	HKAS57692	China	KT990524	KT990728	KT990901	KT990365	Wu et al. 2016
	HKAS89494	China	KT990525	KT990729	KT990902	KT990366	Wu et al. 2016
*A. velutipes*	GDGM52409	China	MH670252	_	_	MH700225	This study
	GDGM44713	China	MH670253	MH700191	MH700213	MH700226	This study
	GDGM42608	China	MH670251	MH700190	MH700212	MN549683	This study
A. cf. venustus	ZhangM142	China	MN204558	MN549668	MN473186	MN549714	This study
	A-2	China	MN204557	MN549679	MN473185	MN549713	This study
	GDGM42800	China	MK123463	_	MN473187	MN549684	This study
*A. venustus*	HKAS82183	China	KU321705	_	_	_	Li et al. 2016
	HKAS77700	China	KU321703	_	_	_	Li et al. 2016
*A. viridiflavus*	DD972	USA	AY612805	_	_	_	GenBank
*A. viscidipes*	GDGM 44818	China	MN204510	_	_	MN549694	This study
	HKAS77103	China	KT990519	KT990723	_	KT990360	Wu et al. 2016
	GDGM44820	China	MK123457	_	_	MN549695	This study
*A. yunnanensis*	GDGM 26359	China	MN204560	MN549670	MN473145	MN549681	This study
	GDGM 24560	China	MN204559	_	MN473144	MN549682	This study
	HKAS57581	China	KF112422	KF112233	KF112560	KF112746	Wu et al. 2016
	HKAS75050	China	KT990520	KT990724	KT990898	KT990361	Wu et al. 2016
*A. zangii*	GDGM 75881	China	MN204563	-	MN473183	MN549712	This study
	GDGM 28577	China	MN204561	_	_	_	This study
	GDGM 44406	China	MN204562	_	MN473151	MN549689	This study
	HKAS63217	China	KT990526	_	_	_	Wu et al. 2016
	HKAS74751	China	KT990521	KT990725	KT990899	KT990362	Wu et al. 2016
	HKAS74766	China	KT990522	KT990726	KT990900	KT990363	Wu et al. 2016
*P. imbricatus*	HKAS68642	China	KF112398	KF112299	KF112637	KF112786	Wu et al. 2014
X. aff. subtomentosus	HKAS58865	China	KF112389	KF112294	KF112630	KF112784	Wu et al. 2014

The combined final dataset was analysed using RAxML v7.2.6 (Stamatakis 2006) and MrBayes v3.1.2 (Ronquist and Huelsenbeck 2003) for Maximum Likelihood (ML) and Bayesian Inference (BI), respectively. For both BI and ML analyses, the substitution model, suitable for each gene partition, was determined using the Akaike Information Criterion (AIC), complemented in MrModeltest v2.3 (Nylander 2004). All parameters in the ML analysis were kept as defaults except for choosing GTRGAMMAI as the model and statistical supports were obtained using rapid non-parametric bootstrapping with 1000 replicates; BI analysis using 4 chains were conducted by setting generations to 80 million and stoprul command with the value of stopval set to 0.01, trees were sampled every 100 generations, the first 25% generations were discarded as burn-ins and posterior probabilities (PP) were then calculated from the posterior distribution of the retained Bayesian trees. *Phylloporus imbricatus* N.K. Zeng, Zhu L. Yang & L.P. Tang and *Xerocomus subtomentosus* (L.) Quél. were selected as outgroups, based on Wu et al. (2016) and Zhang et al. (2017).

## Results

### Molecular phylogenetic results

**Figure 1.**
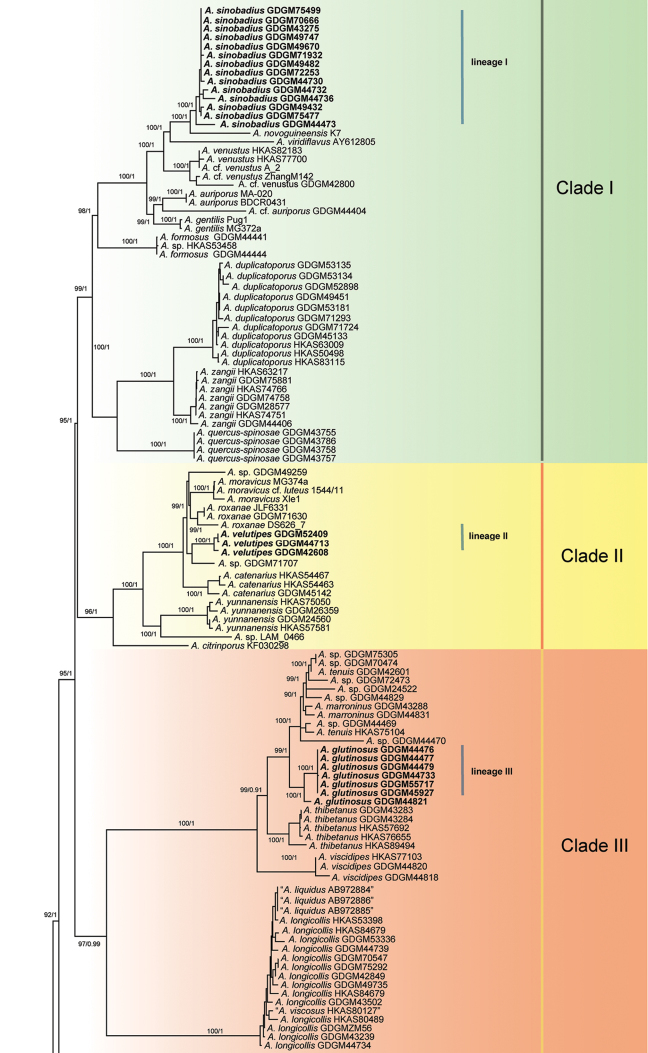
Maximum likelihood tree from a RAXML search using the GTRGAMMA model, illustrating the phylogeny of *Aureoboletus* and related taxa in Boletales, based on a multilocus (nrLSU, *tef1-a*, *rpb1* and *rpb2*) dataset. *Phylloporus imbricatus* N.K. Zeng, Zhu L. Yang & L.P. Tang and *Xerocomus subtomentosus* (L.) Quél. are chosen as outgroups. The lineages with new species and new combination are bold in the tree. Branches are labelled with maximum likelihood bootstrap higher than 70% and Bayesian posterior probabilities more than 0.95.

For phylogenetic analyses, 304 (102 nrLSU, 59 *tef1-a*, 71 *rpb1* and 72 *rpb2*) new sequences from 105 *Aureoboletus* collections and 171 GenBank downloaded sequences from 68 *Aureoboletus* samples were used as ingroups. Four sequences of *P. imbricatus* and *X. subtomentosus*, respectively, retrieved from GenBank were used as outgroups. The combined matrix of 175 samples with 3018 nucleotide sites was submitted to TreeBASE (Submission ID 25249). HKY+G, GTR+I+G, SYM+I and SYM+G were chosen as the best substitution models for nrLSU, *tef1-a*, *rpb1* and *rpb2*, respectively. ML and BI analyses generated almost identical tree topologies with minimal variations in statistical support values. Thus, only a ML tree is displayed (Fig. 1).

In the multi-gene phylogenetic trees, the monophyly of *Aureoboletus* was statistically strongly supported (BS = 100, PP = 1); eight well supported main clades, labelled as Clade I to VIII, are shown and six well supported (BS = 100, PP = 1) new species lineages were recognised. In Clade I, nine known species [*A. auriporus* (Peck) Pouza, *A. duplicatoporus* (M. Zang) G. Wu & Zhu L. Yang, *A. formosus* Ming Zhang & T.H. Li, *A. gentilis* (Quél.) Pouzar, *A. novoguineensis* Hongo, *A. quercus-spinosae* Ming Zhang & T.H. Li, *A. venustus* Fang Li, Kuan Zhao & Qing Li Deng, *A. viridiflavus* Coker & Beers ex Klofac and *A. zangii* X.F. Shi & P.G. Liu] were presented, including the type species *A. gentilis* and a new lineage (lineage I) discovered in this study. Lineage I is presented as a sister group to *A. novoguineensis* with significant statistical support (BS = 100, PP = 1). Clade II comprised five known species [*A. catenarius* G. Wu & Zhu L. Yang, *A. citriniporus* (Halling) Klofac, *A. moravicus* (Vaček) Klofac, *A. roxanae* (Frost) Klofac and *A. yunnanensis* G. Wu & Zhu L. Yang], a new lineage (lineage II) and three unnamed sequences. Lineage II is closely related to an unnamed sample (GDGM71707) from southern China. Clade III is composed of six species, [*A. longicollis* (Ces.) N.K. Zeng & Ming Zhang, *A. marroninus* T.H. Li & Ming Zhang, *A. tenuis* T.H. Li & Ming Zhang, *A. thibetanus* (Pat.) Hongo & Nagas., *A. viscidipes* (Hongo) G. Wu & Zhu L. Yang] and a new lineage (lineage III), all of which are from Asia. Clade IV was comprised of the North American species *A. auriflammeus* (Berk. & M.A. Curtis) G. Wu & Zhu L. Yang and a new species combination from China. Clade V included five strongly supported species level groups [*A. innixus* (Frost) Halling, A.R. Bessette & Bessette, *A. nephrosporus* G. Wu & Zhu L. Yang, *A. rubellus* Kuan Zhao & G. Wu] and two new lineages (lineage IV and lineage V). Clade VI included four known species [*A. mirabilis* (Murrill) Halling, *A. projectellus* (Murrill) Halling, *A. russellii* (Frost) G. Wu & Zhu L. Yang from North America and *A. shichianus* (Teng & L. Ling) G. Wu & Zhu L. Yang from China]. Clade VII had a single species, *A. clavatus* N.K. Zeng & Ming Zhang, which was recently reported in southern China. Clade VIII represents a single new lineage (lineage VI), which is the basal group of the genus *Aureoboletus*.

### Taxonomy

#### Aureoboletus

Taxon classificationFungiBoletalesBoletaceae

Pouzar, Česká Mykol. 11: 48, 1957.

73CC6FFD-0074-5AFA-A9C3-025084834F65

##### Type species

. *Aureoboletus gentilis* (Quél.) Pouzar.

##### Description.

Basidiomata small to large. Pileus viscid, dry or sticky when wet, even or smooth to wrinkled, usually subtomentose, rarely glabrous, with or without veil or velar residues hanging at margin. Context white to yellowish-white, usually pinkish to reddish-brown beneath pileipellis, unchanging or changing blue or greenish or pastel red when exposed. Tubes coloured with all kinds of yellows, pale yellow, golden yellow to bright yellow, unchanging or slightly changing to blue when bruised, pores circular to angular, smaller to larger, somewhat relatively larger and shallowly depressed around the stipe, concolorous with tubes. Stipe central, cylindrical or clavate, surface glabrous to striate fibrillose, never or rare forming reticulation or *Leccinum*-like scabrous, dry to viscid, with white basal mycelium. Basidiospores smooth to verrucose or longitudinally striate, subfusiform, oblong ovoid to subglobose, yellowish to yellowish-brown in KOH. Hymenophoral trama boletoid, composed of subcylindrical to cylindrical hyphae, colourless. Pleurocystidia fusiform to subclavate, thin- or thick-walled, sometimes containing golden-yellow contents at first, then gradually changing to yellowish-white to hyaline in 5% KOH. Cheilocystidia present, infrequent or absent, usually similar to pleurocystidia in shape and size, if present. Pileipellis as an interwoven trichoderm, trichoderm or ixotrichoderm, consisting of erect hyphae which are occasionally branched, cylindrical to clavate, thin- to slightly thick-walled, usually less than 1 μm. Stipitipellis hymeniform, as an ixotrichoderm to intricated ixotrichoderm. Caulocystidia clavate, fusoid or ventricose-fusoid. Stipe trama composed of parallel hyphae. Clamp connections absent.

##### Distribution and ecology.

World-wide distribution, mainly known from subtropical Asia and temperate zones of the Northern hemisphere, growing on the ground associated with Fagaceae and other broadleaf trees.

### Descriptions of six new species and one new combination of *Aureoboletus*

#### Aureoboletus glutinosus

Taxon classificationFungiBoletalesBoletaceae

Ming Zhang & T.H. Li sp. nov.

9D187891-0F47-5FE3-9663-A5E20FEFDB0B

827103

**Figure 2.**
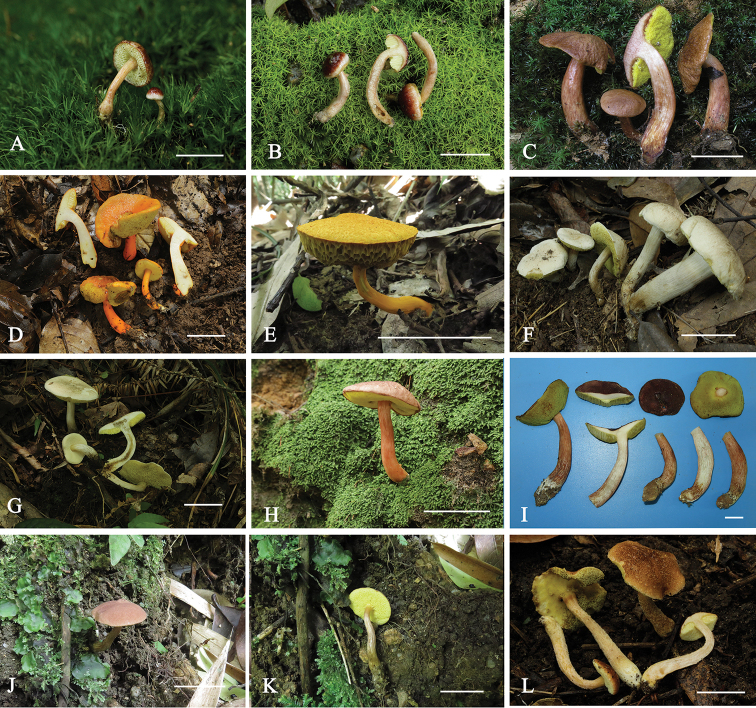
Basidiomata of six new species and one new combination of *Aureoboletus* from China. **A, B***A. glutinosus* (**A** GDGM44476 **B** GDGM44477, holotype) **C***A. griseorufescens* (GDGM28490, holotype) **D, E***A. miniatoaurantiacus* (**D** GDGM43439 **E** GDGM43282) **F, G***A. raphanaceus* (**F** GDGM45911, holotype **G** GDGM52890) **H, I***A. sinobadius* (**H** GDGM44732 **I**GDGM 71932, holotype) **J, K***A. solus* (GDGM44759, holotype) **L***A. velutipes* (**L** GDGM44713, holotype). Scale bars: 2 cm.

**Figure 3.**
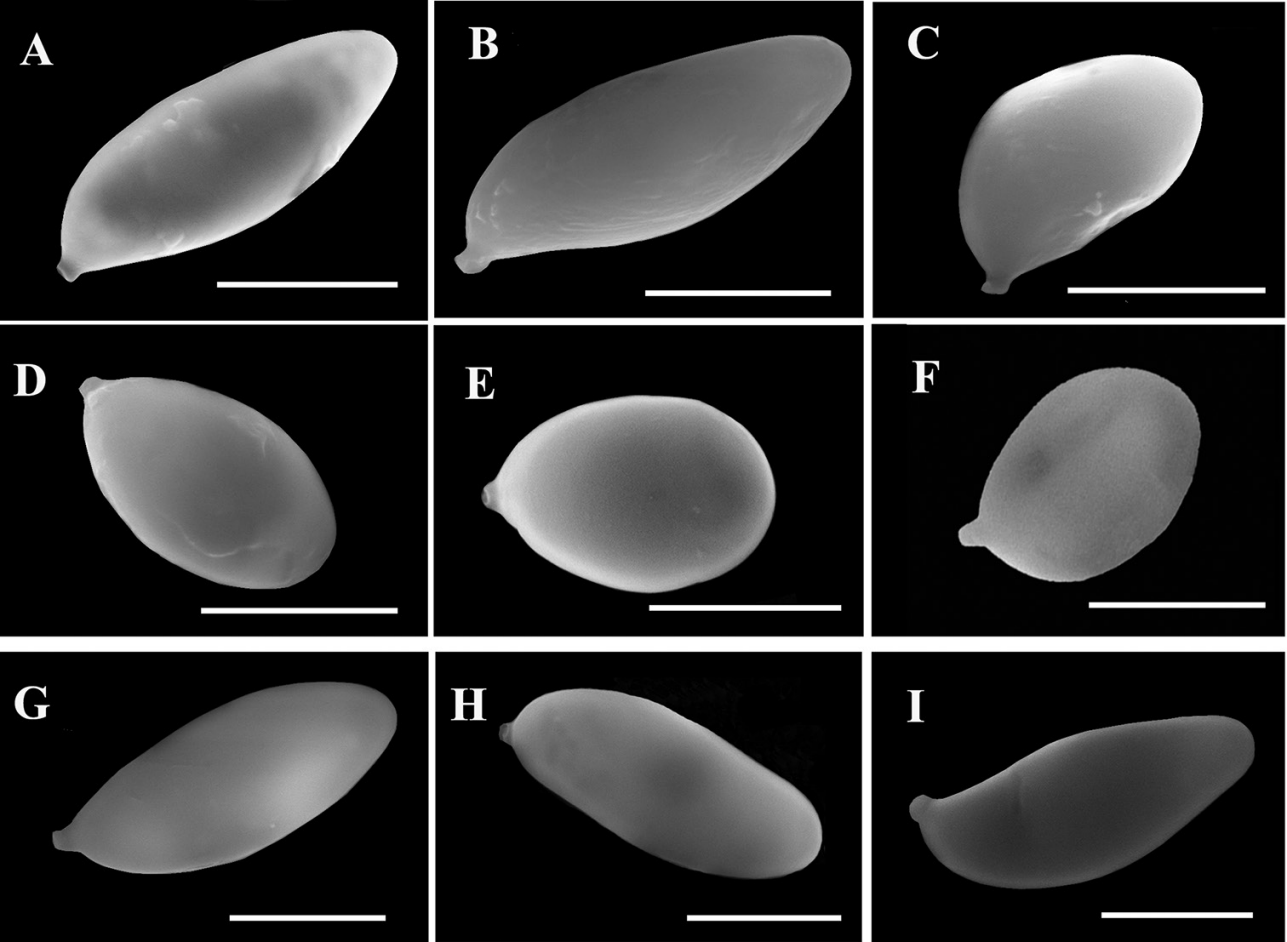
Scanning electron micrograph of basidiospores of six new species and one new combination of *Aureoboletus* from China. **A***A. glutinosus* (GDGM44477, holotype) **B***A. griseorufescens* (GDGM28490, holotype) **C, D***A. miniatoaurantiacus* (**C** GDGM43439 **D** GDGM4855) **E, F***A. raphanaceus* (GDGM45911, holotype) **G***A. sinobadius* (GDGM71932, holotype) **H***A. solus* (GDGM44759, holotype) **I***A. velutipes* (GDGM44713, holotype). Scale bars: 5 µm.

**Figure 4.**
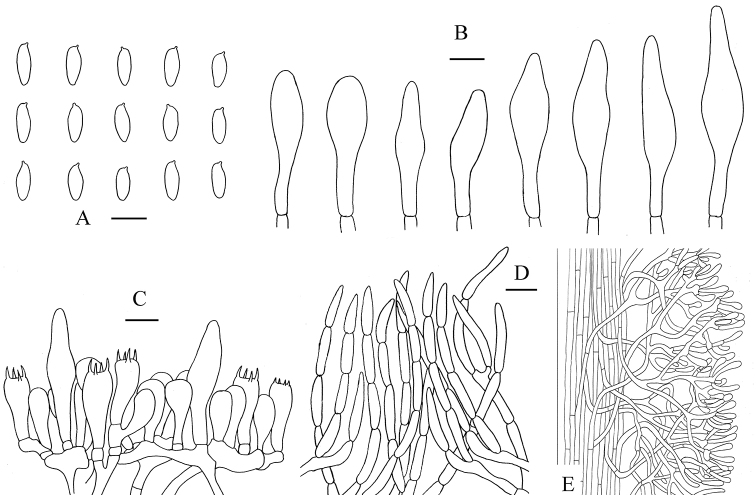
*Aureoboletus glutinosus*. **A** Basidiospores **B** Cheilocystidia and pleurocystidia **C** Basidia and pleurocystidia **D** Pileipellis **E** Stipitipellis. Scale bars: 10 µm (**A–C**); 20 µm (**D, E**).

Figs 2A, B , 3A , 4A–E

##### Diagnosis.

This species is distinguished from other *Aureoboletus* taxa by its smaller and glutinous basidiomata, reddish-brown to ruby pileus usually with irregular reticulation and darker folds, gelatinised veil remnants and smooth basidiospores 10–13.5 × 4.5–5 µm in size.

##### Etymology.

“*glutinosus*” refers to the glutinous basidiomata.

##### Type.

China, Hunan Province, Rucheng Town, Jiulongjiang National Forest Park, on soil and usually growing amongst the mosses under the broadleaf forest, at 25°38'N, 113°77'E, alt. 300 m, 8 May 2014, M. Zhang (holotype: GDGM44477).

##### Description.

Basidiomata small-sized. Pileus 1–2 cm wide, obtuse to convex, becoming broadly convex to plane, fleshy, viscid, especially when young and wet, reddish-brown, violet brown to greyish-ruby (9E6–12E6, 9E7–12E7), slightly fading to pale yellow (2A3–4A3) towards margin, usually forming a pale yellow to even nearly white zone at margin, distinctly wrinkled and often reticulate irregularly with somewhat darker folds at centre, strongly glutinous or mucilaginous when fresh; margin somewhat involute to nearly ﬂat, often attached with yellowish-white to subhyaline and strongly gelatinised veil remnants. Context 2–5 mm thick at stipe, ﬁrm and tough in youth, soft when matured, white on the whole, greyish-red (10B5–11B5) beneath pileipellis, practically unchanging to becoming slightly greyish-pinkish or greyish-red (10B5–11B5) when exposed. Tubes 7–10 mm deep, distinctly depressed around stipe, yellowish-white (2A2–4A2) when young, becoming pale yellow, greyish-yellow, pastel yellow to olive yellow (2A3–4A3, 1B3–2B3, 2A4–3A4, 2C6–3C6) with age, often with an olive tint, unchanging when bruised. Pores 0.3–0.5 mm in diam., mostly subangular, slightly radially elongated around stipe at maturity, smaller near pileus margin, concolorous with tubes. Stipe 15–40 × 2–4 mm, central, cylindrical or narrowly clavate, solid, equal to slightly tender downwards, greyish-orange (6B4), greyish-red (7B4) to brownish-orange (6C4–7C4), without reticulation, smooth to faintly longitudinally striate, gelatinous or strongly viscid when young and wet, usually covered with a mucilaginous layer, with white basal mycelium. Odour not distinct. Taste mild.

Basidiospores [150/4/4] (9.5–)10–13.5 × (4–)4.5–5 µm, Q = (2.2–)2.3–2.5(–2.7), Q_m_ = 2.48 ± 0.18, subfusiform and inequilateral in side view, oblong in ventral view, smooth, yellowish to yellowish-brown in 5% KOH and yellow brown to dark brown in Melzer’s reagent, thin-walled. Basidia 20–30 × 7–10 µm, clavate, 4-spored, sterigmata 2–4.5 µm long, yellowish-white to hyaline in 5% KOH, without basal clamps. Pleurocystidia 35–60 × 8–13 μm, fusiform, thin-walled. Cheilocystidia frequent, similar to pleurocystidia in shape and size. Hymenophoral trama composed of subparallel hyphae 4–10 μm broad, yellowish-white to hyaline in 5% KOH. Pileipellis an ixotrichodermium of erect hyphae 5–12 μm in diameter, branched, yellowish-white to hyaline in 5% KOH, dextrinoid in Melzer’s reagent; terminal cells 27–50 × 7–12 µm, cylindrical, clavate or nearly fusoid. Stipitipellis a layer of repent to suberect branching hyphae 3–6 μm in diam., hyaline in 5% KOH. Clamp connections absent in all tissues.

##### Ecology and distribution.

Solitary or scattered on ground with humus and debris, usually growing amongst the mosses (*Fissidens* sp. and *Pottiaceae* sp.) under Fagaceae, mixed with other broadleaf trees, alt. 300–500 m; May to July, known from Guangdong and Anhui Province.

##### Additional specimens examined.

China, Hunan Province, Chenzhou City, Rucheng Town, Jiulongjian National Forest Park, 8 May 2014, H. Huang (GDGM44476); Same location, 12 June 2015, M. Zhang (GDGM44733); Anhui Province, Huangshan City, Huangshan National Forest Park, 27 July 2015, C.H. Li (GDGM44821).

##### Notes.

Phylogenetic analyses showed that *A. glutinosus* is closely related to *A. marroninus*, *A. tenuis*, *A. thibetanus* and *A. viscidipes*; however, the independent phylogenetic position and different morphological characters can distinguish *A. glutinosus* from these similar species. *Aureoboletus marroninus* differs in having a more wrinkled and darker (violet brown or maroon) pileus, white context and smaller basidiospores 8.5–10 × 4–4.5 µm (Zhang et al. 2014). *Aureoboletus tenuis* has relatively larger basidiomata (pileus up to 3.5 cm broad) usually lacking well-developed veil remnants on pileus margin, smaller basidiospores 11–12 × 4–5 μm and ixotrichodermial stipitipellis composed of terminal hyphae with swollen tips (Zhang et al. 2014). *Aureoboletus thibetanus* is readily separated by its more robust basidiomata (pileus up to 5 cm broad), white ridged reticulation on pileus surface, white stipe and yellowish granular encrustation on cystidia and only known from the temperate zone in southwest China (Patouillard 1895; Yang et al.2003; Klofac 2010). *Aureoboletus viscidipes* differs in having a brownish to brown pileus tinged with yellowish-white, a longer (up to 4 cm long) and nearly white stipe and a thick layer of a reflective pale-yellow substance on the surface of cheilocystidia and pleurocystidia (Wu et al. 2016).

#### Aureoboletus griseorufescens

Taxon classificationFungiBoletalesBoletaceae

Ming Zhang & T.H. Li sp. nov.

FF940C3D-2515-5196-B72D-7FFB38769053

827104

Figs 2C , 3B , 5A–E

##### Diagnosis.

This taxon can be distinguished from other *Aureoboletus* species by its brownish-orange to ruby pileus colour, white to yellowish-white context changing to greyish-red or greyish-rose when exposed, light yellow tubes and comparatively small basidiospores 9–10.5 × 4.5–5 μm.

##### Etymology.

“*griseorufescens*” refers to the greyish-red discolouration of context when exposed or bruised.

##### Type.

China, Guangdong Province, Shaoguan City, Chebaling National Natural Reserve, on soil under the broadleaf forest dominated by Fagaceae trees, alt. 300 m, 23°22'N, 113°42'E, 15 July 2008, C.Y. Deng (holotype: GDGM28490) .

##### Description.

Basidiomata small to medium-sized. Pileus 2–5 cm wide, hemispheric when young, becoming convex to nearly plane in age, fleshy, subviscid or slightly viscid when wet, glabrous to minutely velvet-subtomentose, slightly wrinkled to rugulose, even or nearly so at margin, brownish-orange, brownish-red, dark red to greyish-ruby (6C6–7 to 11C6–7). Context 3–6 mm thick at centre, firm and tough, white to yellowish-white (2A1–2 to 3A1–2), more or less greyish-red (9C4–11C4) beneath the pileipellis and browner at the border line adjacent to tubes, gradually changing to greyish-red (9C4–11C4) to greyish-rose (12B5) when exposed. Tubes 2–4 mm deep, light yellow, yellow, pastel yellow to greenish-yellow (2A5, 3A4–6), unchanging when bruised. Pores small, 1–2 per mm, circular to angular, somewhat relatively larger and shallowly depressed around the stipe at maturity, concolorous with tubes, unchanging when bruised. Stipe 35–60 × 4–10 mm, central, cylindrical or clavate, equal to slightly enlarged downwards, smooth, viscid in wet condition, concolorous with pileus, pale in the apex. Stipe context white to reddish-white (9A2–11A2), gradually changing to greyish-red (9C4–11D5) to greyish-rose (12B5) when exposed, especially in the lower part. Basal mycelium white. Odour none. Taste mild.

Basidiospores [50/2/2] (8–)9–10.5(–11) × (4–)4.5–5(–5.5) μm, Q = (1.8–)2–2.2 (2.6), Q_m_ = 2.19 ± 0.18, subfusiform and inequilateral in side view, oblong in ventral view, smooth, yellowish to yellowish-brown in 5% KOH and yellow brown to dark brown in Melzer’s reagent, thin-walled. Basidia 4-spored 25–30 × 7–11 μm, clavate, yellowish-white to hyaline in 5% KOH, sterigmata 2–3 μm. Cheilocystidia infrequent. Pleurocystidia 43–70 × 8–13 μm, fusiform, thin-walled, yellowish-white to hyaline in 5% KOH. Hymenophoral trama composed of subparallel hyphae 5–8 μm broad, yellowish-white to hyaline in 5% KOH. Pileipellis an entangled trichodermium of erect hyphae 12–19 μm in diameter, branched, yellowish-white to hyaline in 5% KOH, yellow brown to dark brown in Melzer’s reagent, terminal cells 20–50 × 6–10 μm, cylindrical, clavate or nearly fusoid. Stipitipellis a tangled layer of repent to suberect branching hyphae 7–10 μm in diam., hyaline in 5% KOH, with terminal cells 22–30 × 7–18 μm. Caulocystidia 43–58 × 12–18 μm, numerous, in clusters, clavate, fusoid or fusoid ventricose, mostly clavate, swollen at apex and usually contain yellow to yellowish-brown substance at an early stage in 5% KOH. Clamp connections absent in all tissues.

##### Ecology and distribution.

Solitary or scattered on ground with humus and debris under Fagaceae trees, mixed with other broadleaf trees, alt. 200–400 m; June to September; currently only known from southern China.

##### Additional specimens examined.

China, Hainan Province, Changjiang County, Bawangling National Forest Park, 7 July 2013, M. Zhang (ZhangM131).

##### Notes.

*Aureoboletus griseorufescens* is somewhat similar to the recently reported species *A. venustus* from southern China; however, the latter taxon differs in having relatively larger (pileus up to 8 cm) and more viscous basidiomata, a reddish-orange pileus and broader basidiospores 7.5–10.5 × 5–6 μm (Li et al. 2016). In addition, *A. griseorufescens* formed a separate species level branch at the base of the phylogenetic tree (Fig. 1), indicating that it is in an independent phylogenetic position.

**Figure 5.**
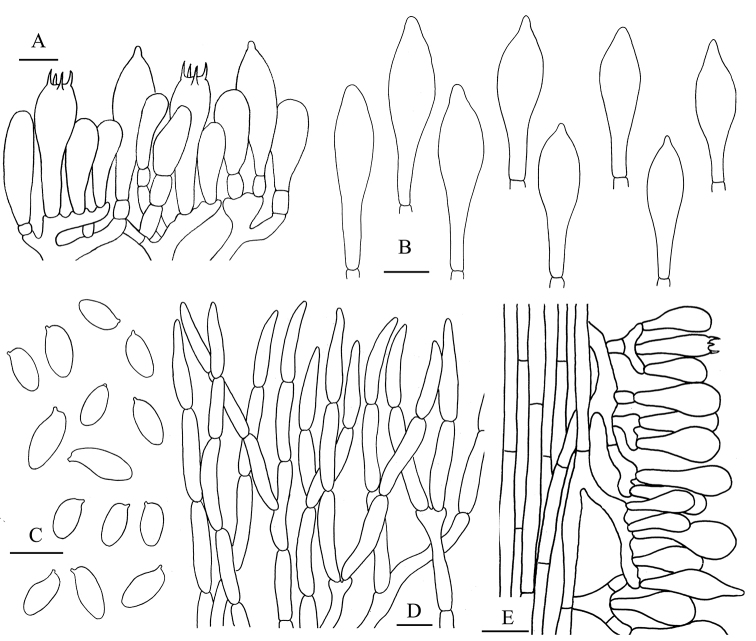
*Aureoboletus griseorufescens*. **A** Basidia and pleurocystidia **B** Cheilocystidia and pleurocystidia **C** Basidiospores **D** Pileipellis **E** Stipitipellis. Scale bars: 10 µm (**A–C**); 20 µm (**D, E**).

#### Aureoboletus raphanaceus

Taxon classificationFungiBoletalesBoletaceae

Ming Zhang & T.H. Li sp. nov.

D9AAE876-F609-5B0B-874E-9DE9A689B919

827106

**Figure 6.**
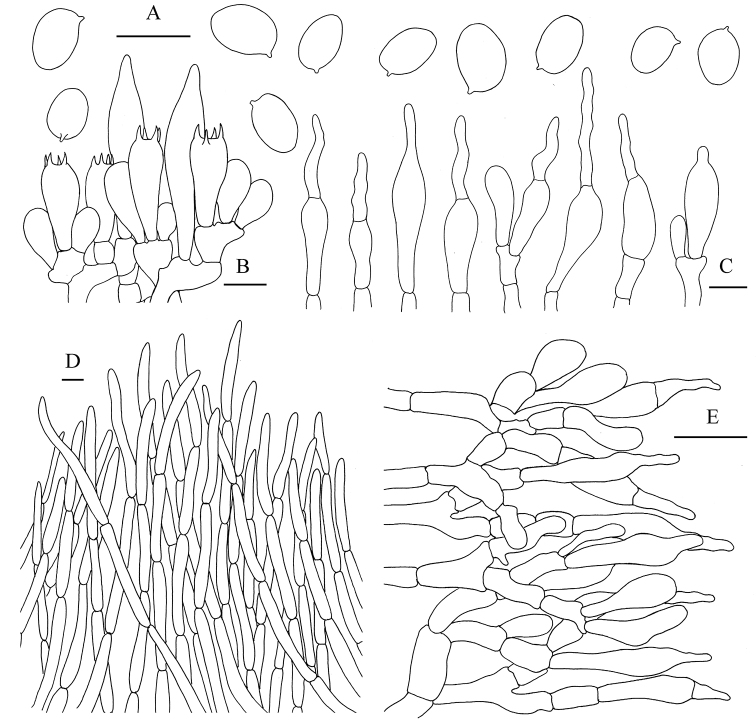
*Aureoboletus raphanaceus*. **A** Basidiospores **B** Basidia and pleurocystidia **C** pleurocystidia **D** Pileipellis **E** Stipitipellis. Scale bars: 10 µm (**A–C**), 20 µm (**D, E**).

Figs 2F, G , 3E, F , 6A–E

##### Diagnosis.

This species can be easily distinguished from other *Aureoboletus* taxa by its dry and yellowish-white to pinkish-white pileus covered with fibrillose to tomentose squamules, radish smell and ovoid basidiospores 7.5–9 × 5–6 μm.

##### Etymology.

“*raphanaceus*” refers to the radish smell of the new species.

##### Type.

China, Jiangxi Province, Chongyi Town, Yangling National Forest Park, on soil under the broadleaf forest dominated by Fagaceae trees, at 25°28'N, 114°19'E, alt. 300 m, 1 September 2016, H. Huang (holotype: GDGM45911).

##### Description.

Basidiomata small to medium-sized. Pileus 3–8 cm wide, hemispheric when young, becoming convex to nearly plane in age, fleshy, dry or slightly viscid when wet, covered with greenish-grey, yellowish-grey to brownish-grey (1D2–10D2) fibrillose to tomentose squamules on yellowish-white (1A2–4A2) to pinkish-white background, slightly wrinkled at disc; margin thin, slightly incurved at first, then extending. Context 8–12 mm thick at centre, firm and tough in youth, becoming soft, white, more or less pinkish, brownish-orange (5C4–7C4), greyish-red (8C4–10C4) to light brown (5D4–7D4) beneath the pileipellis, unchanging or slightly changing blue near the hymenophore when exposed. Tubes 4–7 mm deep, greyish-yellow (1B5–3B5), light yellow (1A5–3A5) to yellow (2A7–3A7), unchanging when bruised. Pores small, 0.5–1 per mm, circular to angular, somewhat relatively larger and shallowly depressed around the stipe at maturity; pore-surface concolorous with tubes, unchanging when hurt. Stipe 20–40 × 8–15 mm, central, cylindrical or clavate, equal to slightly enlarged downwards, dry, concolorous with pileus, longitudinally streaked and faintly pruinose or tomentose, with a very pale flush of pastel red (8A5–10A5) zone at apex. Stipe context white to yellowish-white, slightly changing pale yellow (2A3–4A3) when exposed, especially in the lower part. Basal mycelium white. Odour as radish. Taste mild.

Basidiospores [80/3/3] (7–)7.5–9(–10) × 5–6 μm, Q= (1.27–)1.45–1.6(–1.7), Q_m_ = 1.51 ± 0.08, ovoid and inequilateral in side view, ovoid in ventral view, smooth, yellowish to pale brown in 5% KOH and yellowish-brown in Melzer’s reagent, thin-walled. Basidia 20–30 × 8–11 μm, clavate, 4-spored, rarely 1-, 2-, 3-spored, yellowish-white to hyaline in 5% KOH, without basal clamps, sterigmata 2–3.5 µm long. Pleurocystidia 30–60 × 8–13 μm, fusiform, thin-walled, usually containing golden-yellow contents at first, gradually changing yellowish-white to hyaline in 5% KOH. Cheilocystidia infrequent, similar to pleurocystidia in shape and size. Hymenophoral trama composed of subparallel hyphae 5–23 μm broad, yellowish-white to hyaline in 5% KOH. Pileipellis an ixotrichodermium to trichodermium of erect hyphae 4–12 μm in diameter, usually covered with yellow to brownish-yellow pigment slightly dissolving in 5% KOH, branched, yellowish-white to hyaline in 5% KOH, dextrinoid in Melzer’s reagent; terminal cells cylindrical, clavate or nearly fusoid. Stipitipellis a layer of suberect branching hyphae 4–15 μm in diameter, hyaline in 5% KOH. Caulocystidia 30–60 × 8–12 μm, numerous, in clusters, fusiform to lageniform and usually contain yellow to yellowish-brown substance in an early stage in 5% KOH. Clamp connections absent in all tissues.

##### Ecology and distribution.

Solitary or scattered on ground with humus and debris under Fagaceae trees mixed with other broadleaf trees, alt. 300–1300 m; June to September; Currently known from Jiangxi and Hunan Province.

##### Additional specimens examined.

China, Jiangxi Province, Chongyi County, Yangling National Forest Park, alt. 550 m, 1 September 2016, M. Zhang (GDGM52908); Same locality and date B. Song (GDGM53127), M. Zhang (GDGM52266 and GDGM50266), H Huang (GDGM52890); Hunan Province, Guidong Town, Bamianshan National Nature Reserve, alt. 1250 m, 18 June 2016, Z.P. Song (GDGM52543 and GDGM46333).

##### Notes.

The yellowish-white basidioma colour makes it easy to distinguish *A. raphanaceus* from the other species. *Boletus orientialbus* N.K. Zeng & Zhu L. Yang recently described from China is somewhat similar to *A. raphanaceus* in colour; however, *B. orientialbus* differs in having more robust basidiomata, smooth pileus, reticulate stipe and smaller basidiospores 7–10 × 4.5– 5 μm (Zeng et al. 2013).

#### Aureoboletus sinobadius

Taxon classificationFungiBoletalesBoletaceae

Ming Zhang & T.H. Li sp. nov.

F6262386-5D2A-536B-941A-B0B53283B1A0

827101

**Figure 7.**
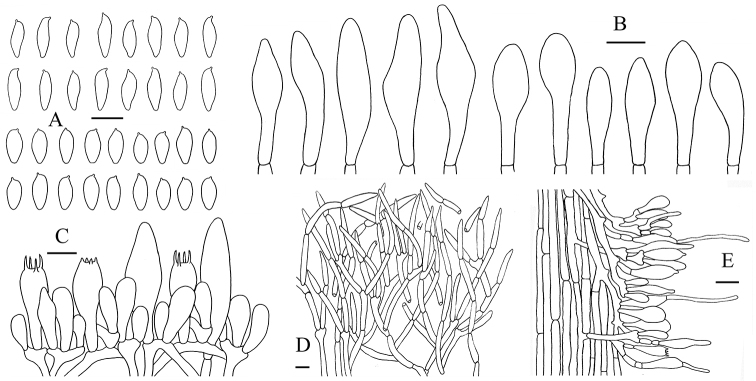
*Aureoboletus sinobadius*. **A** Basidiospores **B** Cheilocystidia and pleurocystidia **C** Basidia and pleurocystidia **D** Pileipellis **E** Stipitipellis. Scale bars: 10 µm (**A–C**); 20 µm (**D, E**).

Figs 2H, I , 3G , 7A–F

##### Diagnosis.

This species is distinguished from other *Aureoboletus* species by its pastel red to reddish-brown pileus, light yellow hymenophore unchanging when bruised, salty taste and two different shapes of basidiospores.

##### Etymology.

“*sino*-” refers China, the holotype’s location of the species; “*badius*” means the brownish-red or chestnut pileus colour.

##### Type.

China, Guangdong Province, Guangzhou City, Baiyun Mountain Scenic Area, on soil and usually growing amongst moss under broadleaf forest, dominated by Fagaceae trees, alt. 280 m, 18 May 2018, M. Zhang (holotype: GDGM71932).

##### Description.

Basidiomata medium to large-sized. Pileus 5–10 cm wide, hemispheric when young, becoming convex to nearly plane in age, fleshy, viscid, especially when young and wet, glabrous to minutely velvet-subtomentose, slightly wrinkled, usually violet brown (10E5–8 to 12E5–8) when young, gradually fading to pastel red (8A5–10A5), brownish-red (9C7–10C7), reddish-brown to brownish-violet (9D6–11D6, 9D7–11D7) at maturity, with a thin and slightly incurved margin. Context 7–10 mm thick at centre, firm and tough in youth and later soft, white to yellowish-white, and more or less greyish-red (9C4–10C4) beneath the pileipellis, slightly changing to greyish-red (9C4–10D5) when exposed. Tubes 8–15 mm deep, light yellow to greenish-yellow (2A5, 2B5), unchanging when bruised. Pores small, 1–1.5 per mm, circular to angular, somewhat relatively larger and shallowly depressed around the stipe at maturity, unchanging when bruised; pore-surface concolorous with tubes. Stipe 40–80 × 5–9 mm, central, cylindrical or clavate, equal to slightly enlarged downwards, smooth, viscid when wet, pastel red (8A5–10A5), with a very pale flush of pale orange (5A3–6A3) fibrous stripe. Stipe context white to yellowish-white, slightly changing to greyish-red (9C4–10D5) when bruised. Basal mycelium white. Odour mild. Taste salty.

Basidiospores [150/8/5] 10–13(–14) × (4–) 4.5–5 (–5.5) μm, average 11.5–12.5 × 4.5–5, Q = (–2) 2.3–2.67 (–2.88), Q_m_ = 2.44 ± 0.22, subfusiform and inequilateral in side view with an obtuse apex, oblong to ovoid in ventral view, smooth, yellowish to yellowish-brown in 5% KOH, yellow brown to dark brown in Melzer’s reagent, occasionally two different shapes in some specimens. Basidia 22–33 × 8–11 μm, clavate, predominantly 4-spored, partially 2-spored, with sterigmata 2–4 µm long, yellowish-white to hyaline in 5% KOH, without basal clamp. Pleurocystidia 27–50 × 7–13 μm, fusiform, thin-walled, usually containing golden-yellow contents at first, gradually changing from yellowish-white to hyaline in 5% KOH. Cheilocystidia frequent, 23–48 × 9–15 μm, clavate to subfusiform, thin-walled, containing golden-yellow contents at first, gradually changing yellowish-white to hyaline in 5% KOH. Hymenophoral trama composed of subparallel hyphae 4–10 μm broad, yellowish-white to hyaline in 5% KOH. Pileipellis an ixotrichodermium of erect and branched hyphae 6–12 μm in diameter, yellowish-white to hyaline in 5% KOH, dextrinoid in Melzer’s reagent; terminal cells 35–60 × 5–10 μm, cylindrical, clavate or nearly fusoid. Stipitipellis a layer of repent to suberect branched hyphae 3–10 μm in diam., hyaline in 5% KOH. Caulocystidia 30–45 × 9–18 μm, mostly swollen clavate, usually containing yellow to yellowish-brown substance at an early stage in 5% KOH. Clamp connections absent in all tissues.

##### Ecology and distribution.

Solitary or scattered on ground with humus and debris under *Castanopsis fissa* Rehder E.H. Wilson mixed with other broadleaf trees, alt. 200–300 m; known from south China.

##### Additional specimens examined.

China, Guangdong Province, Guangzhou City, Baiyun Mountain Scenic Area, alt. 300 m, 4 June 2015, M. Zhang (GDGM44736 and GDGM44732); Same location, alt. 300 m, 30 May 2013, M. Zhang (GDGM43275); Same location, alt. 300 m, 4 June 2013, M. Zhang (ZhangM55); Same location, alt. 280 m, 14 May 2015, M. Zhang (GDGM45920); Guangdong Province, Huizhou City, Xiangtoushan National Nature Reserve, alt. 300 m, 2 April 2015, M. Zhang (GDGM44473); Hunan Province, Chenzhou City, Jiulongjiang National Forest Park, alt. 280 m, 13 June 2015, M. Zhang (GDGM44730); Guangzhou City, Research Institute of Tropical Forestry, alt. 200 m, 4 May 2018, J. Xu (GDGM72253).

##### Notes.

*Aureoboletus sinobadius* is morphologically similar to *A. auriporus*, *A. flaviporus* (Earle) Klofac, *A. gentilis*, *A. novoguineensis* and *A. venustus*. However, *A. auriporus* differs from *A. sinobadius* in the pinkish cinnamon, vinaceous to vinaceous brown pileus, longer and more robust stipe covered with yellow pruina or floccosity at apex, slight acid taste and broader basidiospores 11–16 × 4–6 μm (Pouzar 1957; Smith and Thiers 1971; Halling 1989; Both 1993; Bessette et al. 2000; Klofac 2010); *A. flaviporus* differs in the pale cinnamon to dark reddish-brown pileus, reddish-brown stipe usually with reticulation at the apex, acidic taste, broader basidiospores 11–15 × 4–6 μm and the known distribution in North America (Bessette et al. 2000); *A. gentilis*, originally described from Europe, differs in having pinkish-brown to flesh-coloured pileus, whitish context unchanging when exposed and longer and broader basidiospores 12–15 × 5–6.5 μm (Singer 1945; Pouzar 1957; Klofac 2010); *A. novoguineensis*, originally described from New Guinea, has pale pink brown or pale red context, shorter (3–4 mm deep) and sometimes compound hymenophore, acid taste and larger basidiospores (11.5–15.5 × 4.5–5.5 μm) and pleurocystidia (36–66 × 13–18 μm) (Hongo 1973); *A. venustus* recently described from southern China differs by its shorter and broader basidiospores 7.5–10.5 × 5–6 μm (Li et al. 2016).

#### Aureoboletus solus

Taxon classificationFungiBoletalesBoletaceae

Ming Zhang & T.H. Li sp. nov.

D60072D7-6C09-50A3-9CD1-9AAC32D1E877

827105

Figs 2, K, 3H, 8A–D

##### Diagnosis.

This species can be easily distinguished from other *Aureoboletus* taxa by its dry and small basidiomata, brownish-yellow to greyish-red pileus, glabrous stipe and smaller basidiospores (7–)8–10.5(–11) × (4–)4.5–5 μm.

##### Etymology.

“*solus*” refers to the solitary habit.

##### Type.

China, Guangdong Province, Shaoguan City, Nanling National Nature Reserve, on soil under the broadleaf forest, dominated by Fagaceae trees, 16 June 2015, M. Zhang (holotype: GDGM44759).

##### Description.

Basidiomata small-sized. Pileus 1.5–2.5 cm wide, hemispheric when young, becoming convex to nearly plane in age, fleshy, dry or slightly viscid when wet, minutely velvet subtomentose, slightly wrinkled, brownish-yellow, brownish-orange, brownish-red to greyish-red (5C7–8C7, 5C5–9C5); margin thin, slightly incurved at first, becoming nearly straight, often appendiculate with small membranous remains of the veil. Context 2–6 mm thick at centre, firm and tough in youth, becoming soft, white, more or less greyish-red (9C5–11C5) to brownish-red (9C7–11C7) beneath the pileipellis, unchanging when exposed. Tubes 2–3 mm deep, gr-yish-yellow (1B5–3B5), light yellow (1A5–3A5) to vivid yellow (1A8–3A8), gradually changing to greenish-yellow when mature, unchanging when bruised, shallowly depressed around the stipe at maturity. Pores small, 1–2 per mm, somewhat larger around the stipe, circular to angular; pore-surface concolorous with tubes. Stipe 20–45 × 2–6 mm, central, cylindrical or clavate, equal to slightly enlarged downwards, glabrous, dry or slightly viscid when wet, pale orange to pale red (5A3–7A3), pastel red (8A5–10A5), with very pale flush of pastel red (8A5–10A5) fibrous stripes. Stipe context white to pastel red (8A4–10A4), slightly darker when bruised, especially in the lower part. Basal mycelium white. Odour none. Taste mild.

Basidiospores [80/3/3] (7–)8–10.5(–11) ×(4–)4.5–5 μm, Q = (1.5–)1.8–2.2(–2.6), Q_m_ = 2.0 ± 0.21, subfusiform and inequilateral in side view, oblong to ovoid in ventral view, smooth, yellowish to yellowish-brown in 5% KOH and yellow brown to dark brown in Melzer’s reagent, thin-walled. Basidia 1, 2, 4-spored 25–46 × 9–16 μm, clavate, yellowish-white to hyaline in 5% KOH; sterigmata 2–4.5 µm long. Pleurocystidia frequent, 38–66 × 11–15 μm, fusiform, thin-walled, yellowish-white to hyaline in 5% KOH. Cheilocystidia similar to pleurocystidia in shape and size. Hymenophoral trama composed of subparallel hyphae 5–11 μm broad, yellowish-white to hyaline in 5% KOH. Pileipellis an entangled trichodermium of erect hyphae 5–17 μm in diameter, branched, yellowish-white to hyaline in 5% KOH, dextrinoid in Melzer’s reagent; terminal cells cylindrical, clavate or nearly fusoid. Stipitipellis a layer of repent hyphae 4–23 μm in diameter, hyaline in 5% KOH. Caulocystidia infrequent. Clamp connections absent in all tissues.

##### Ecology and distribution.

Solitary or gregarious on soil under broadleaf forests dominated by *Castanopsis* spp. and *Cyclobalanopsis* spp. and mixed with other broadleaf trees, alt. 300–1200 m; May to July, currently only known from Guangdong Province.

##### Additional specimens examined.

China, Guangdong Province, Shaoguan City, Nangling National Nature Reserve, alt. 1200 m, 29 July 2017, M. Zhang (GDGM70342); Guangdong Province, Huizhou County, Xiangtoushan National Nature Reserve, alt. 400 m, 16 June 2016, J.P. Zou (GDGM46222); Guangdong Province, Huizhou City, Nankunshan Provincial Nature Reserve, alt. 700 m, 15 May 2013, M. Zhang (GDGM42822); Guangdong Province, Shaoguan City, Danxianshan National Nature Reserve, alt. 300 m, 3 June 2017, M. Zhang (GDGM46807), Same locality, 2 June 2017, M. Zhang (GDGM49404).

##### Notes.

*Aureoboletus solus* looks like *A. tenuis*; however, the latter differs from the former in its viscid basidiomata, ixotrichodermial stipitipellis, composed of terminal hyphae with slightly swollen tips and larger basidiospores (10–)11–12 × 4–5 µm (Zhang et al. 2014). Phylogenetic analyses indicated that *A. solus* is closely related to *A. nephrosporus*, but *A. nephrosporus* differs in having larger basidiomata with a red to brownish-red pileus, ovoid to nephroid basidiospores 8–10.5 × 5–6 µm and cheilocystidia and pleurocystidia covered with a thick layer of a strongly refractive pale yellow substance (Wu et al. 2016).

**Figure 8.**
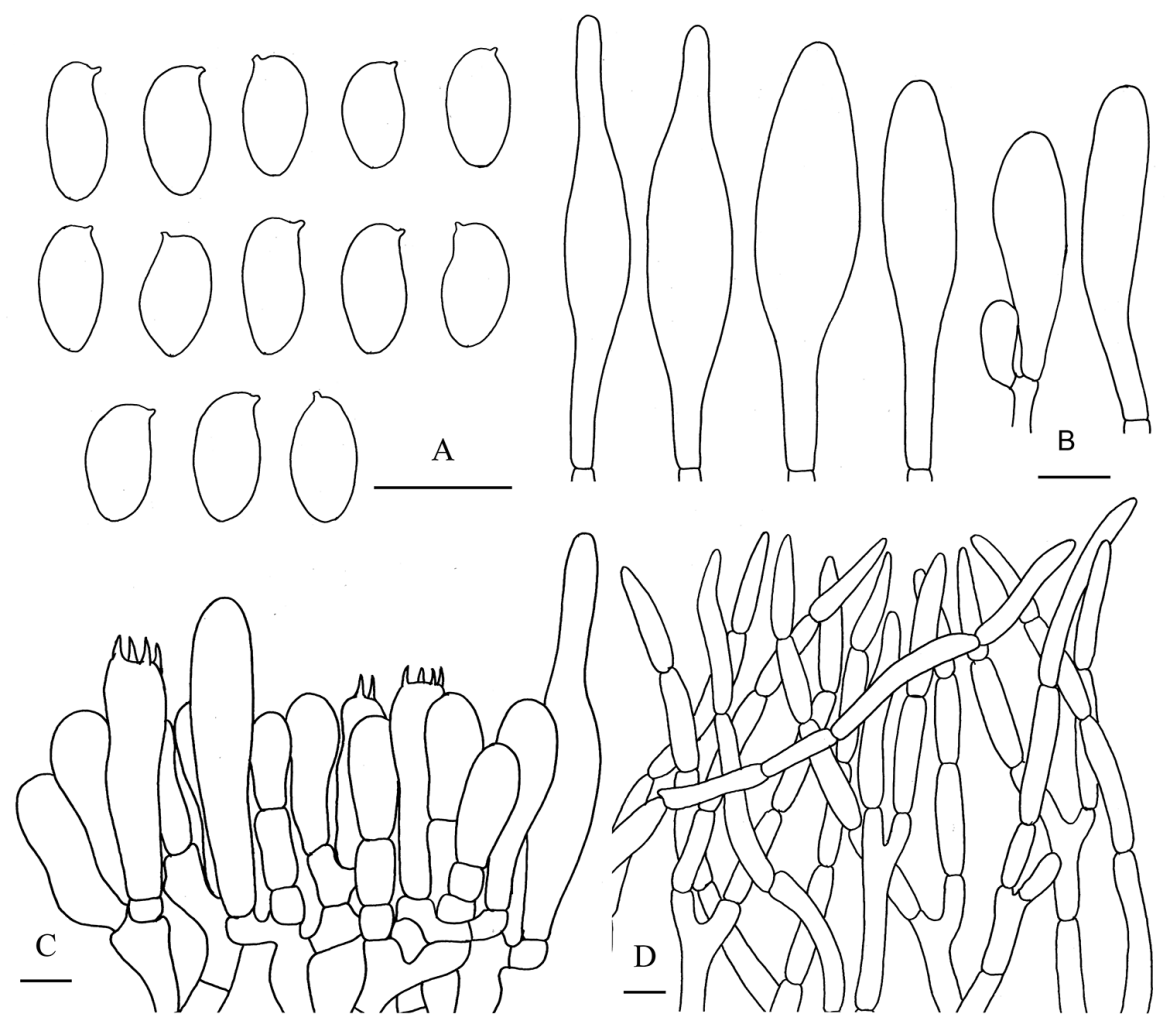
*Aureoboletus solus*. **A** Basidiospores **B** Cheilocystidia and pleurocystidia **C** Basidia and pleurocystidia **D** Pileipellis. Scale bars: 10 µm (**A–D**).

#### Aureoboletus velutipes

Taxon classificationFungiBoletalesBoletaceae

Ming Zhang & T.H. Li sp. nov.

6E1F09EA-3BCB-5E27-B813-C27A1A3716A5

827108

Figs 2L , 3I , 9A–E

##### Diagnosis.

This species can be easily distinguished from others in *Aureoboletus* by its dry and small basidiomata, brown orange to reddish-brown pileus, light yellow to pastel yellow stipe, covered with fibrillose to tomentose squamules and smooth basidiospores 10–13 × 4–6.5 μm.

##### Etymology.

“*velutipes*” refers to the stipe, covered with fibrillose to tomentose squamules.

##### Type.

China, Guangdong Province, Huizhou City, Xiangtoushan National Nature Reserve, on soil under the broadleaf forest, dominated by Fagaceae trees, alt. 350 m, 2 April 2015, M. Zhang (holotype: GDGM44713).

Basidiomata small-sized. Pileus 2–4 cm wide, obtuse to convex when young, becoming broadly convex to plane at mature, fleshy, dry, covered with fibrillose to tomentose squamules, light yellow, light orange (4A4–6A4), brownish-orange (6C7–7C7), brown to reddish-brown (6D7–9D7), slightly fading to light orange to brownish-orange towards margin. Context 3–5 mm thick at stipe, ﬁrm and tough in youth, soft when matured, yellowish to white on the whole, more or less reddish-brown beneath the pileipellis, slightly changing to pastel red (7A4–9A4) when exposed. Tubes 3–5 mm deep, distinctly depressed around stipe, yellowish-white (2A2–4A2) when young, becoming pale yellow, greyish-yellow, pastel yellow to olive yellow (2A3–4A3, 1B3–2B3, 2A4–3A4, 2C6–3C6) in age, often with an olive tint, unchanging when bruised. Pores 0.5–0.8 mm in diam., mostly subangular, slightly elongated around stipe at maturity, smaller near pileus margin, concolorous with tubes. Stipe 30–60 × 5–10 mm, central, cylindrical or narrowly clavate, solid, equal to slightly enlarged downwards, covered with white, yellowish-white to yellowish-brown fibrillose to tomentose squamules, usually forming reticulation or longitudinally striate, light yellow to pastel yellow (2A4–4A4, 2A5–4A5), with white basal mycelium. Odour none. Taste mild.

Basidiospores [90/3/3] 10–13 × (4–)5–6(–6.5) μm, Q = (1.75–)1.8–2.2(–2.4), Q_m_ = 2.08 ± 0.35, subfusiform and inequilateral in side view, oblong to ovoid in ventral view, smooth, yellowish to yellowish-brown in 5% KOH and yellow brown to dark brown in Melzer’s reagent, thin-walled. Basidia 25–30 × 9–13 μm, clavate, predominantly 4-spored but frequently also 2-spored, with sterigmata 2–3 µm long, yellowish-white to hyaline in 5% KOH, without basal clamps. Pleurocystidia 35–65 × 10–18 μm, fusiform, thin-walled. Cheilocystidia frequent, similar to pleurocystidia in shape and size. Hymenophoral trama composed of subparallel hyphae 6–10 μm broad, yellowish-white to hyaline in 5% KOH. Pileipellis a trichodermium of erect and often branched hyphae 4–17 μm in diameter, yellowish-white to hyaline in 5% KOH, dextrinoid in Melzer’s reagent; terminal cells 30–60 × 4–17 μm, cylindrical, clavate or nearly fusoid. Stipitipellis a layer of repent to suberect branching hyphae 3–15 μm in diameter, with swollen tips, terminal cells 30–70 × 11–21 μm, hyaline in 5% KOH. Clamp connections absent in all tissues.

##### Ecology and distribution.

Scattered on soil in subtropical forests, dominated by Fagaceae (*Castanopsis* spp., *Lithocarpus* spp. and *Quercus* spp., etc). Currently known from southern China.

##### Additional specimens examined.

China, Guangxi Province, Guilin City, Maoershan National Nature Reserve, alt. 1380 m, 1 July 2012, M. Zhang (GDGM42608); Jiangxi Province, Jinggangshan City, Jingganshan National Nature Reserve, alt. 1000 m, 21 June 2016, H. Huang (GDGM52409).

##### Notes.

The obviously villose or fibrous squamulose stipe can distinguish it from other species in *Aureoboletus*. *Aureoboletus catenarius*, recently described from southwest China, is somewhat similar to *A. velutipes* with a dry and tomentose pileus, but *A. catenarius* has a cracked and light brown to reddish-brown pileus, faintly or ﬁnely ﬁbrillose stipe and smaller basidiospores 7–9 × 3.5–5 μm (Wu et al. 2016).

**Figure 9.**
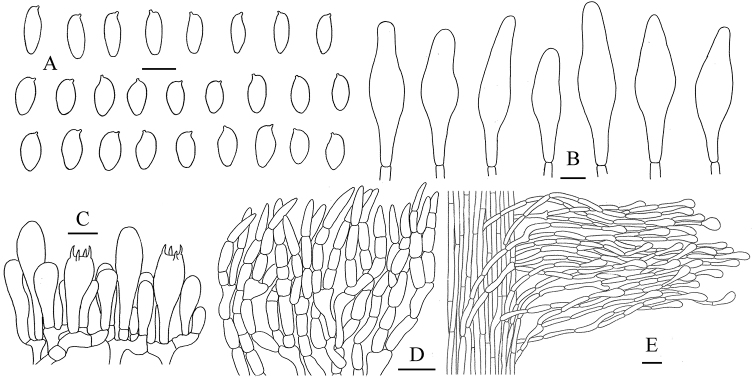
*Aureoboletus velutipes*. **A** Basidiospores **B** Pleurocystidia **C** Basidia and pleurocystidia **D** Pileipellis **E** Stipitipellis. Scale bars: 10 µm (**A–C**), 40 µm (**D, E**).

#### Aureoboletus miniatoaurantiacus

Taxon classificationFungiBoletalesBoletaceae

(Bi & Loh) Ming Zhang, N.K. Zeng & T.H. Li comb. nov.

E871E3AD-BA00-5BD6-9F27-4A5C0092A2F0

827109

**Figure 10.**
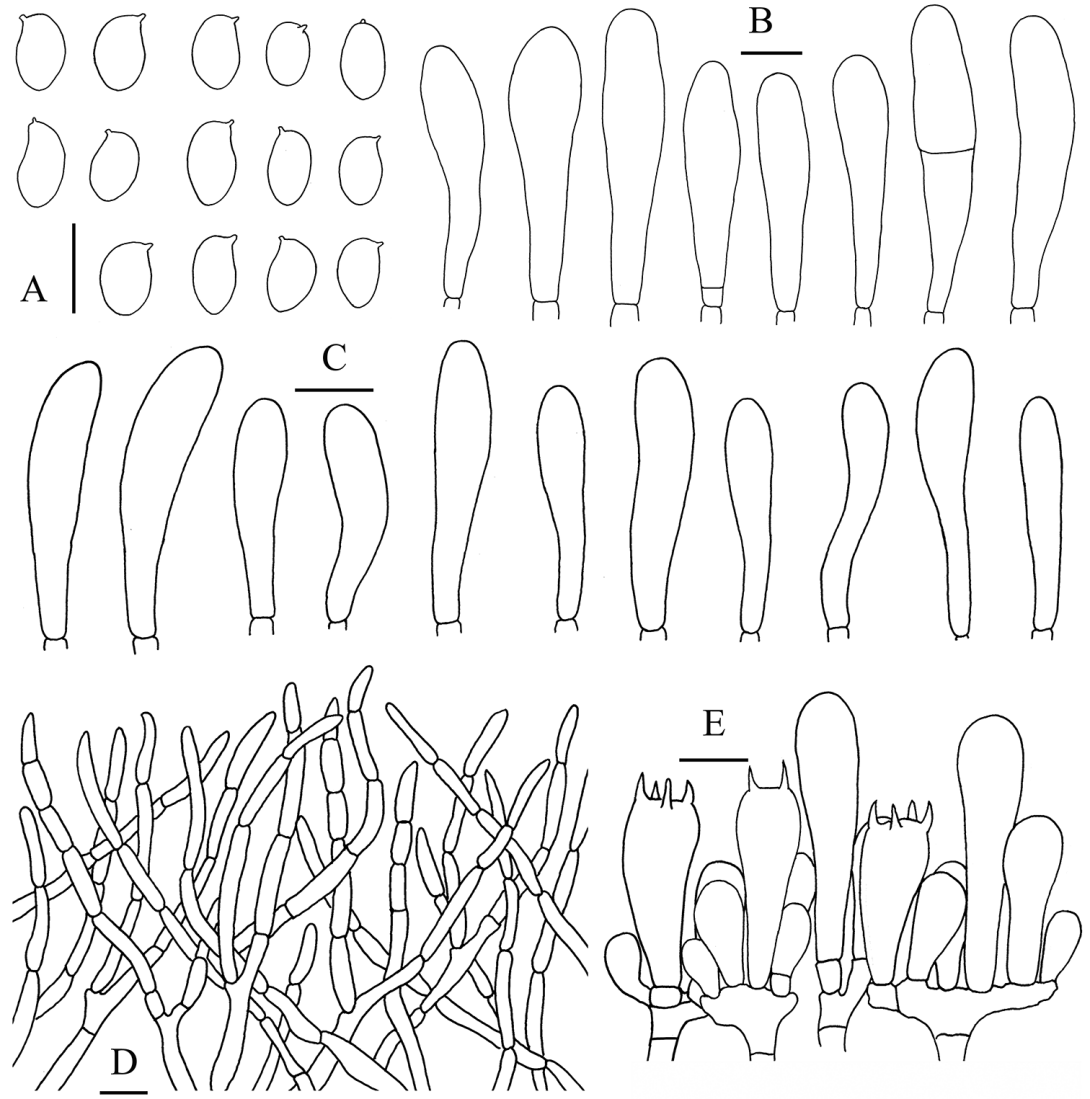
*Aureoboletus miniatoaurantiacus*. **A** Basidiospores **B** Cheilocystidia **C** Pleurocystidia **D** Basidia and pleurocystidia **E** Pileipellis. Scale bars: 10 µm (**A–D**), 20 µm (**E**).

Figs 2D, E , 3C, D , 10A–D

##### Basionym.

*Boletus miniatoaurantiacus* C.S. Bi & Loh, in Bi, Loh & Zheng, Acta Bot. Yunn. 4(1): 60, 1982

##### Synonym.

*Aureoboletus tomentosus* G. Wu & Zhu L. Yang, in Wu, Li, Zhu, Zhao, Han, Cui, Li, Xu & Yang, Fungal Diversity 81: 51, 2016

##### Diagnosis.

In Bi et al. (1982): Pileus 1–1.6 cm latus, siccus, obtuse hemisphaerius, aurantiacus, confertim et minute villoso-tomentosus. Contexto flava, immutibili, ad stipitem 2–3 cm crasso, sapor mitis et odor nullus. Stipes centralis, 3–3.3 cm longus, 3–6 mm crassus, albidus, in parte in feriore flavus, subcylindraceus, solidus, velutinus. Tubuli albidi, immutabiles, ad stipitem breviter decurrentes, 3 mm longi, facile denudati; pori ovati, majuscules, 3 mm diam. Sporae ellipsoideae, laeves, pallido-flavae, 7–10 × 3.3–4 μm, 1 guttatae. Pleurocystidiis 35 × 6.5 μm, paucis.

Basidiomata small to medium-sized. Pileus 1.5–8 cm wide, hemispheric when young, becoming convex to nearly plane in age, fleshy, dry or viscid when wet, surface minutely tomentose or pulverous, slightly wrinkled, orange yellow, reddish-yellow, orange to reddish-orange (4A6–7A6, 4A7–7A7), commonly with a thin and slightly extended margin. Context 5–10 mm thick at centre, firm and tough in youth and, later, soft, white to yellowish-white, with more or less green tint at border contacting tubes, unchanging when exposed. Tube 3–10 mm deep, light orange to orange unchanging when bruised. Pores polygonal, 0.5–1.5 per mm, somewhat relatively larger and shallowly depressed around the stipe, orange to pale orange unchanging when bruised. Stipe 30–80 × 4–10 mm, central, solid, cylindrical or clavate, equal to slightly enlarged downwards, smooth to distinctly longitudinally streaks or broad reticulations, viscid in wet condition, concolorous with pileus. Stipe context concolorous with that of pileus, unchanging when exposed. Basal mycelium white to yellowish-white. Odour strong. Taste mild.

Basidiospores [90/3/3] (6.5–)7–10.5(–11) × (4–)4.5–5.5(–6) μm, Q = (1.42–)1.6–2.0(–2.3), Q_m_ = 1.79 ± 0.18, ovoid and inequilateral in side view with an obtuse apex, ovoid in ventral view, smooth, yellowish to yellowish-brown in 5% KOH and yellow brown to dark brown in Melzer’s reagent, thin-walled. Basidia 18–35 (45) × 7–14 μm, 4-spored, rarely 1-, 2-, 3-spored, clavate, yellowish-white to hyaline in 5% KOH, sterigmata 2–3 μm. Cheilocystidia (21) 26–55 (61) × (6) 8–12 μm, fusiform to subclavate, thin-walled, contained with bright yellow pigments. Pleurocystidia similar to cheilocystidia in shape and size, thin-walled, yellowish-white to hyaline in 5% KOH. Hymenophoral trama composed of interwoven branched hyphae 6–15 μm wide, yellowish-white to hyaline in 5% KOH. Pileipellis an entangled trichodermium to ixotrichodermium of erect hyphae 4–18 μm in diameter, composed of yellow to bright yellow vacuolar pigmented filamentous hyphae, terminal cells cylindrical, clavate or nearly fusoid. Stipitipellis a tangled layer of repent to suberect branching hyphae 7–12 μm in diameter, hyaline in 5% KOH. Caulocystidia 25–75 × 12–18 μm, common, clavate, fusoid or fusoid ventricose and usually contain yellow to yellowish-brown substance in an early stage in 5% KOH. Stipe trama composed of parallel hyphae 4–18 μm wide. Clamp connections absent in all tissues.

##### Ecology and distribution.

Scattered on soil in tropical to subtropical forests dominated by Fagaceae (*Castanopsis chinensis*, *C. fissa*, *Lithocarpus* spp. and *Quercus* spp.). Currently known from southern and southwest China

##### Additional specimens examined.

China, Guangdong Province, Zhaoqing City, Dinghu Mountain, 6 September 1980, C.S. Bi et al. 677 (GDGM4677, holotype of *B. miniatoaurantiacus*); Same locality, 14 April 1981, C. Li (GDGM5071); 11 August 1981, C.S. Bi et al. 855 (GDGM4855); Fujian Province, Zhangping City, alt. 350 m, 2 September 2009, N.K. Zeng 664, 669 (FHMU424, 429); same locality, 27 July 2013, N.K. Zeng 1294 (FHMU848); 29 July 2013, N.K. Zeng 1323 (FHMU876); 1 August 2013, N.K. Zeng 1339 (FHMU891); Guangdong Province, Guangzhou City, Tianluhu Forest Park, alt. 200 m, 29 May 2015, M. Zhang (GDGM42855); Guangdong Province, Shaoguan City, Chebaling National Nature Reserve, alt. 300 m, 3 September 2013, M. Zhang & C.Q. Wang (GDGM43282); Guangdong Province, Huizhou City, Xiangtoushan National Nature Reserve, alt. 300 m, 7 July 2015, M. Zhang (GDGM44727); Jiangxi Province, Chongyi County, Yangling National Forest Park, alt. 280 m, 14 August 2015, M. Zhang (GDGM51694 and GDGM43439); same locality, 31 August 2016, H. Huang (GDGM52888); Same locality, 1 September 2016, M. Zhang (GDGM53350); Same locality, 2 September 2016, M. Zhang (GDGM53274); Same locality, 3 September 2016, M. Zhang (GDGM53501).

##### Notes.

*Aureoboletus miniatoaurantiacus*, originally described as *B. miniatoaurantiacus*, is a rather common species in southern China and can be easily distinguished by its bright orange-yellow basidiomata, tomentose or pulverulent pileus surface, light orange to orange hymenophore unchanging when bruised and ovoid basidiospores. Based on a re-study of the type specimen and other collections quoted by Bi et al. in 1994, we found that the type specimen is composed of two small immature basidiomata, which are in a poor condition for morphological observation, but other voucher specimens fit well with the description of *A. tomentosus*. Thus, the newly described species *A. tomentosus* is, in fact, a synonym of *A. miniatoaurantiacus*, this conclusion also being supported by molecular data in this study (Bi et al. 1982; Bi et al. 1994; Wu et al. 2016). *Aureoboletus auriflammeus*, originally described from North America, is similar to *A. miniatoaurantiacus*; however, the former differs in having a distinctly reticulate stipe and narrower basidiospores (8–12 × 3–5 μm) (Murrill 1908; Bessette et al. 2000).

### Key to the species of *Aureoboletus* known in China

**Table:** 

1	Pileus dry or slightly viscid when wet	**2**
–	Pileus viscid	**13**
2	Basidiomata medium to larger (≥ 6 cm in diam.)	**3**
–	Basidiomata smaller (< 6 cm in diam.)	**4**
3	Pileus 6–10 cm in diameter, greyish-orange to brownish-orange; stipe glabrous, greyish-yellow on the upper part to blond on the lower part; context unchanging when cut; basidiospores 9–11 × 4–5.5 µm	***A. yunnanensis***
–	Pileus 6–15 cm in diameter, brownish-red to reddish-brown; context yellowish-white changing to yellowish-olivaceous when injured; hymenophore pale yellow to olivaceous yellow; stipe surface with longitudinal stripe, brownish-red to reddish-brown; basidiospores subglobose, 7–8 × 5.5–6 µm	***A. clavatus***
4	Stipe surface smooth or with small dots or splotches	**5**
–	Stipe surface non-smooth, with reticula, longitudinal stripe, flocci or others	**11**
5	Basidiospores nodulose to verrucose, 12–15 × 8–11 µm; basidiomata small, golden brown to umber; stipe up to 7 cm long	***A. shichianus***
–	Basidiospores surface smooth; other characters not as above	**6**
6	Hymenophore bright yellow to vivid yellow, unchanging when old	**7**
–	Hymenophore pale yellow, light yellow, greenish-yellow to olive brown	**8**
7	Pileus greyish-rose to brownish-red, glabrous to slightly subtomentose; context pale yellow to light yellow unchanging when cut; stipe dark orange to yellow ochre, with distinct longitudinal streaks and furfuraceous scales; basidiospores 8–10.5 × 5–6 µm	***A. nephrosporus***
–	Pileus reddish-brown to greyish-ruby, smooth to minutely velvet-subtomentose; context white to yellowish-white changing to greyish-red to greyish-rose when exposed; stipe smooth, concolorous with pileus; stipe context white to reddish-white gradually changing to greyish-red to greyish-rose when bruised; basidiospores 9–10.5 × 4.5–5 μm	***A. griseorufescens***
8	Pileus yellowish-white to reddish-white; context white, unchanging or slightly changing blue near the hymenium when exposed; tubes greyish-yellow to light yellow, unchanging when bruised; stipe context white to yellowish-white, slightly changing pale yellow when exposed; odour with radish scent; basidiospores 5–9 × 5–6 μm	***A. raphanaceus***
–	Pileus coloured with brownish-red to reddish-brown tonalities	**9**
9	Pileipellis epithelium; basidiospores subfusoid, 7–9 × 3.5–5 μm	***A. catenarius***
–	Pileipellis trichoderm	**10**
10	Basidiospores fusiform to ovoid, 8–10.5 × 4.5–5 μm	***A. solus***
–	Basidiospores oblong to ovoid, 8.5–10.5 × 5–5.5 µm	***A. rubellus***
11	Stipe surface ornamented with distinctly reticulation, pileus surface covered with coarse tomentose, basidiospores (20–)22–27(–28) × 9–13 µm	***A. mirabilis***
–	Stipe surface without reticulation or the reticulation inconspicuous	**12**
12	Stipe surface ornamented with distinctly longitudinally streaks or dotted scales; pileus surface orange yellow, reddish-yellow to reddish-orange, covered with tomentose; hymenophore light orange to orange; basidiospores 7–11 × 4.5–6 μm	***A. miniatoaurantiacus***
–	Stipe surface covered with distinctly fibrillose to tomentose squamules; pileus surface brownish-orange to reddish-brown, covered with fibrillose to tomentose squamules; context yellowish-white changing to pastel red when exposed; basidiospores 10–13 × 4–6.5 μm	***A. velutipes***
13	Pileus margin with a gelatinised membranous veil	**14**
–	Pileus margin without any membranous veil	**19**
14	Pileus surface distinctly reticulate, coarsely rugose, chestnut-brown to pale brown; stipe whitish; pleurocystidia covered with yellow substance on surface; basidiospores 9.5–13 × 4.5–5 µm	***A. thibetanus***
–	Pileus glabrous or slightly rugose in central	**15**
15	Basidiospores smooth	**16**
–	Basidiospores longitudinally costate, 12–16 × 9–12 µm	***A. longicollis***
16	Pileus brownish to brown; basidiospores 10–12.5 × 4.5–5 µm	***A. viscidipes***
–	Pileus reddish-brown to violet brown	**17**
17	Basidiomata usually ≥ 2.5 cm; basidiospores 11–13.5 × 4.5–5.5 µm	***A. tenuis***
–	Basidiomata usually < 2.5 cm	**18**
18	Basidiospores 8.5–10 × 4–4.5 µm	***A. marroninus***
–	Basidiospores 10–13.5 × 4.5–5 µm	***A. glutinosus***
19	Pileus wrinkled, greyish-yellow to brownish-orange; taste salty; distribution in subalpine zone, ectomycorrhizal with *Quercus spinose*; basidiospores 15–21 × 5–6.5 μm	***A. squercus-spinosae***
–	Pileus smooth, fibrillose to tomentose; the rest of the characters usually not as above	**20**
20	Basidiospores relatively broader, 7.5–10.5 × 5–6 μm, Q_m_ < 2	***A. venustus***
–	Basidiospores relatively narrower, usually Q_m_ ≥ 2	**21**
21	Basidiospores comparatively larger, 15–16.5 × 4.5–5 μm	***A. formosus***
–	Basidiospores comparatively smaller, commonly < 15 μm long	**22**
22	Basidiomata small to medium-sized (pileus usually < 5 cm). Pileus yellowish-brown or reddish-golden, subtomentose; stipe light brown to brownish-orange; basidiospores 9–11 × 4–5 μm	***A. zangii***
–	Basidiomata medium to large (pileus usually > 5 cm)	**23**
23	Pileus violet brown to brownish-violet, glabrous to minutely velvet-subtomentose; stipe pastel red with a pale flush fibrous stripe; taste salty; basidiospores 10–14 × 4.5–5.5 μm	***A. sinobadius***
–	Pileus reddish-brown to brownish-red, nearly glabrous; stipe reddish-orange to garnet brown with faintly longitudinal streaks; taste unknown, basidiospores 8.5–13 × 4.5–5.5 μm	***A. duplicatoporus***


## Discussion

### Species delimitation, species diversity and new taxa in China

In the taxonomic circumscription of the genus *Aureoboletus* proposed by Pouzar (1957), 35 species were identified prior to this study, of which 20 species were recorded from China (i.e. *A. auriporus*, *A. catenarius*, *A. clavatus*, *A. duplicatoporus*, *A. formosus*, *A. longicollis*, *A. marroninus*, *A. mirabilis*, *A. nephrosporus*, *A. quercus-spinosae*, *A. rubellus*, *A. shichianus*, *A. tenuis*, *A. thibetanus*, *A. tomentosus*, *A. venustus*, *A. viscidipes*, *A. viscosus*, *A. yunnanensis* and *A. zangii*). However, the report of the North American species *A. auriporus* was excluded from China in this study due to a misidentification, as its correct name is *A. sinobadius*. The previously described species, *A. tomentosus*, was proven to be *A. miniatoaurantiacus* and so, a new combination is proposed here. Six species, *A. glutinosus*, *A. griseorufescens*, *A. sinobadius*, *A. solus*, *A. raphanaceus* and *A. velutipes*, obtained from China, are newly described in this study.

The present study demonstrates that species of *Aureoboletus* are very diverse in China, especially in its southern areas. Common morphological characteristics and molecular data make *Aureoboletus* easily distinguishable from other existing genera in Boletaceae, but some variable morphological features make it difficult to recognise some species. Careful examination showed that several morphological characteristics are available to delimit these species in China. For example, the colour of the hymenophore and pattern of the pileus are important characteristics: *A. glutinosus* has a light yellow to olive yellow hymenophore and a coarse pileus with irregular reticulation; *A. sinobadius* has a vivid yellow hymenophore and a subtomentose to glabrous and viscid pileus; *A. miniatoaurantiacus* has a light orange to orange hymenophore and a tomentose to pulverous pileus; regarding the size of basidiomata, *A. clavatus* and *A. yunnanensis* have relatively larger basidiomata up to 10 cm in diameter, whereas *A. glutinosus* and *A. marroninus* have smaller basidiomata usually less than 2.5 cm in diameter. The colour of the pileus and the colour and odour of the context also help to identify species in the field. In contrast to macro-morphology, several micro-morphological features can also be used to discriminate species of *Aureoboletus*, such as the size and shape of basidiospores and the shape and inclusion of cystidia, pileipellis and stipitipellis seem to be rather constant amongst the different species.

### Phylogenetic analyses supported the presence of eight clades in *Aureoboletus*

In the present study, all selected samples of *Aureoboletus* formed a well-supported monophyletic group and eight major clades are proposed here, based on morphological characteristics and phylogenetic inference.

Clade I is characterised by the presence of a viscid pileus, a vivid yellow to greyish-yellow hymenophore that is unchanging when bruised, smooth basidiospores and ixotrichodermium pileipellis. In the present study, this group contains ten species, including the type species *A. gentilis* and the new species *A. sinobadius*. This clade is a rather homogeneous group in terms of morphology, which is consistent with the definition of *Aureoboletus* given by Pouzar (1957). Species in this clade can be separated from each other by pileus colour and the size of basidiospores. In addition, two unsequenced species, *A. flavimarginatus* and *A. flaviporus*, should belong to this clade, based on their morphological characteristics (viscid pileus and vivid yellow hymenophore).

Clade II is characterised by the presence of a dry (or slightly sticky when wet) pileus, a vivid yellow to olive yellow hymenophore that is unchanging when bruised, smooth basidiospores and trichodermium pileipellis. This clade includes six species, of which *A. velutipes* has distinctive morphological characteristics, such as a villous pileus and stipe, pale yellow to olive yellow hymenophore and swollen tips in terminal cells of the stipitipellis.

Clade III is well-characterised by the presence of a viscid pileus with well-developed yellowish to subhyaline veil remnant at the margin, greyish-yellow to olive yellow hymenophore, smooth to longitudinally costate basidiospores and ixotrichodermium pileipellis. *Aureoboletus longicollis*, originally described from Malaysia, is a well-defined species in this clade and is readily distinguished by its more viscid and larger basidioma, longer stipe and longitudinally costate basidiospores. A Chinese species, *A. viscosus*, shares similar traits with *A. longicollis* and the two species cannot be separated from each other in morphology. In this study, we did not have access to specimens of *A. longicollis* from Malaysia for morphological and phylogenetical study and it is not possible to make a taxonomic decision on whether *A. viscosus* is the same or a different species to *A. longicollis* without phylogenetic data. Thus, the name *A. longicollis* is temporarily used in this study and further studies with more materials are needed. The other species in this clade are characterised by smooth basidiospores and they can be distinguished from each other by their pileus colour and the size of basidiospores.

Clade IV contains the species *A. auriflammeus* and *A. miniatoaurantiacus*, which are mainly characterised by their bright orange yellow basidiomata, tomentose pileus surface and ovoid basidiospores. Species in this clade can be easily distinguished from others in this genus.

Clade V is characterised by the presence of a dry or somewhat tacky pileus, greyish-yellow to vivid yellow hymenophore changing to olive yellow when mature and oblong, ovoid to nephroid basidiospores. This clade contains five species, including the two species *A. solus* and *A. raphanaceus* described above.

Clade VI is composed of four distinct species, which have all been recently added to *Aureoboletus*, based on phylogenetic analyses (Halling et al. 2015, Wu et al. 2014, 2016). *Aureoboletus projectellus*, *A. mirabilis* and *A. russellii* were originally described from North America and have a dry or coarsely tomentose pileus, distinct coarse reticulations on the stipe and larger basidiospores (up to 20 μm); however, the basidiospores of *A. projectellus* and *A. mirabilis* are smooth, while *A. russellii* has longitudinally costate basidiospores (Murrill 1938; Singer 1945; Smith and Thiers 1971; Pegler and Young 1981; Bessette et al. 2000). *A. shichianus*, originally described from southwest China, is a remarkable species in this clade and differs from the others by its small basidiomata, tomentose pileus, radially arranged pores, comparatively long and glabrous stipe and scabrous basidiospores with nodules. Species in this clade are quite diverse, though the coarsely reticulated stipe and ornamented basidiospores are unique in the genus.

Clade VII is currently formed by a single species, *A. clavatus*. The most striking characteristics are the large basidiomata with slightly viscid pileus, yellowish-white context staining yellowish-olivaceous when exposed, pale yellow to olivaceous-yellow hymenophore, subglobose basidiospores and the pileipellis composed of a turf of clavate hyphae. Besides the slightly viscid pileus, this species shares nearly none of the basic morphological traits of the genus *Aureoboletus*. However, phylogenetic analyses showed that it belongs to *Aureoboletus* and formed a separate branch.

Clade VIII is formed by a single species *A. griseorufescens*. Morphologically, *A. griseorufescens* is similar to those species in Clade I with a vivid yellow hymenophore and subviscid pileus; however, the most striking characteristic of *A. griseorufescens* is its white to yellowish-white context changing to greyish-red or greyish-rose when exposed. In the phylogenetic tree, *A. griseorufescens* formed the basal branch of *Aureoboletus* with highly-supported values, which showed that it might be an early divergent species from *Aureoboletus*.

### Geographical distribution and species evolution

*Aureoboletus* is a cosmopolitan genus, but most known species have relatively distinct habitats or regional locations. Currently, most of known *Aureoboletus* species are distributed in East Asia (mainly in China) and North America and intercontinentally-distributed species are infrequent. Two species *A. projectellus* and *A. mirabilis*, originally reported from North America, were examined in several studies and found to have disjunctive distributions in the North Temperate region from North America to Asia (China, Japan) and Europe (Hongo 1973, Chen et al. 1988, Motiejūnaitė et al. 2013, Halling et al. 2015, Wu et al. 2016). In Asia and North America, some morphologically similar and phylogenetically related species also exist. For example, *A. auriporus* and *A. viridiflavus* are similar to *A. sinobadius* and *A. formosus* and *A. auriflammeus* is similar to *A. miniatoaurantiacus*. However, they can be clearly separated from each other by molecular data. In Europe, only *A. gentilis* was originally described and not found in other continents; this represents a separate geographical region of *Aureoboletus*.

Phylogenetic analyses based on 144 collections uncovered some useful information regarding the geography of *Aureoboletus*. Species in Clades III, V and VIII are found in Asia (China-Japan-Malaysia-Vietnam), representing subtropical-tropical Asia distributions. Compared with North America and Europe, China has the greatest number of *Aureoboletus* species and endemic species, especially in the subtropical-tropical region. Furthermore, many regions in China are under-sampled and more under-described indigenous *Aureoboletus* species will undoubtedly be discovered in the future. The high diversity of *Aureoboletus* species in China indicates that the subtropical-tropical region of China (or Asia) is the current species diversity centre of *Aureoboletus*.

In this study, some evolutionary patterns of morphological characteristics were also discovered. The traits of dry or viscid pileus surface and hymenophore colour appear to be relatively stable evolutionary characteristics and were wellsupported by monophyletic clades on the phylogenetic tree. The shape and surface ornamentation of basidiospores are not reliable characteristics for delimiting *Aureoboletus*, but are useful for species identification. Basidiospores ornamentation may have evolutionarily originated several times within *Aureoboletus* history. More morphological and molecular data are needed to understand this trait.

## Supplementary Material

XML Treatment for Aureoboletus

XML Treatment for Aureoboletus glutinosus

XML Treatment for Aureoboletus griseorufescens

XML Treatment for Aureoboletus raphanaceus

XML Treatment for Aureoboletus sinobadius

XML Treatment for Aureoboletus solus

XML Treatment for Aureoboletus velutipes

XML Treatment for Aureoboletus miniatoaurantiacus
